# Economics of field size and shape for autonomous crop machines

**DOI:** 10.1007/s11119-023-10016-w

**Published:** 2023-04-09

**Authors:** A. K. M. Abdullah Al-Amin, James Lowenberg‑DeBoer, Kit Franklin, Karl Behrendt

**Affiliations:** 1 Harper Adams University, Shropshire, Newport, TF10 8NB UK; 2 Bangladesh Agricultural University, Mymensingh, 2202 Bangladesh

**Keywords:** Swarm robotics, Field size and shape, Field efficiency, Economic feasibility, Economies of size, Profitability

## Abstract

Field size and shape constrain spatial and temporal management of agriculture with implications for farm profitability, field biodiversity and environmental performance. Large, conventional equipment struggles to farm small, irregularly shaped fields efficiently. The study hypothesized that autonomous crop machines would make it possible to farm small, non-rectangular fields profitably, thereby preserving field biodiversity and other environmental benefits. Using the experience of the Hands Free Hectare (HFH) demonstration project, this study developed algorithms to estimate field times (h/ha) and field efficiency (%) subject to field size and shape in grain-oil-seed farms of the United Kingdom using four different equipment sets. Results show that field size and shape had a substantial impact on technical and economic performance of all equipment sets, but autonomous machines were able to farm small 1 ha rectangular and non-rectangular fields profitably. Small fields with equipment of all sizes and types required more time, but for HFH equipment sets field size and shape had least impact. Solutions of HFH linear programming model show that autonomous machines decreased wheat production cost by €15/ton to €29/ton and €24/ton to €46/ton for small rectangular and non-rectangular fields respectively, but larger 112 kW and 221 kW equipment with human operators was not profitable for small fields. Sensitivity testing shows that the farms using autonomous machines adapted easily and profitably to scenarios with increasing wage rates and reduced labour availability, whilst farms with conventional equipment struggled. Technical and economic feasibility in small fields imply that autonomous machines could facilitate biodiversity and improve environmental performance.

**Supplementary Information:**

The online version contains supplementary material available at 10.1007/s11119-023-10016-w.

## Introduction

Field size and shape have substantial consequences for environmental management (Bacaro et al., 2015; Clough et al., 2020; Konvicka et al., 2016; Marja et al., 2019), technical (Fedrizzi et al., 2019; Griffel et al., 2018, 2020; Islam et al., 2017; Janulevičius et al., 2019; Luck et al., 2011) and economic feasibility (Batte & Ehsani, 2006; Carslaw, 1930; Larson et al., 2016; Miller et al., 1981; Sturrock et al., 1977). To facilitate conventional agricultural mechanization, comparatively large rectangular fields are needed and most of the land consolidation around the world in the last decades have been motivated by the desire for larger fields (Kienzle et al., 2013; Van den Berg et al., 2007). Field size and shape has been a key factor in determining international crop competitiveness. Since the advent of motorized mechanization countries with relatively large, roughly rectangular fields have had a major economic advantage (e.g., USA, Canada, Australia, Brazil, Argentina). In the United Kingdom, field size has increased through removing hedgerows and in field trees to allow use of larger machinery and ensure economies of size (MacDonald & Johnson, 2000; Pollard et al., 1968; Robinson & Sutherland, 2002). On the contrary, small fields are often neglected and considered as non-economic. For instance, in the United States many small irregular-shaped fields were abandoned in the twentieth Century. The European Union and Switzerland retained small fields in production with subsidies (Lowenberg-DeBoer et al., 2021a, 2021b; OECD, 2017).

Nevertheless, under the umbrella of landscape management, small fields are promoted by researchers. Research in Canada and the United States found higher biodiversity in smaller fields (Fahrig et al., 2015; Flick et al., 2012; Lindsay et al., 2013). Likewise, studies in the United Kingdom and the European Union also showed that small fields and more fragmented landscapes have higher biodiversity (Firbank et al., 2008; Gaba et al., 2010; González-Estébanez et al., 2011). Using the context of the agricultural low lands of England, Firbank et al. (2008) pointed out that the pressure on biodiversity may be reduced through minimizing habitat loss in agricultural fields. The German case study found that East Germany's large-scale agriculture reduced biodiversity while small-scale agriculture of West Germany had higher biodiversity (Batáry et al., 2017). As the environmental benefits of small fields are well documented in research, it would be interesting to explore the economics of small fields to better identify the win–win scenarios for small fields. Consequently, this study hypothesized that autonomous crop machines would make it possible to farm small, non-rectangular fields profitably, thereby preserving field biodiversity and other environmental benefits.

Autonomous crop machines in this study refer to the mechatronic devices which have autonomy in operation usually through a predetermined field path. More specifically, the autonomous machines are mobile, having decision making capability, and accomplish arable farm operations (i.e., drilling, seeding, spraying fertilizer, fungicide and herbicide, and harvesting) under the supervision of humans, but without the involvement of direct human labour and operator (Lowenberg-DeBoer et al., 2020). Autonomous machines are precision agriculture technology because they have the potential to cost effectively increase the precision of input applications and to collect very detailed data on agricultural production. The autonomous machines, demonstrated by the HFH project used swarm robotics concepts in which multiple smaller robots are used to accomplish farm work usually done by larger conventional machines with human operators. The autonomous swarm robotics of the HFH project are developed by retrofitting conventional diesel operated machines (Hands Free Hectare (HFH), 2021).

Autonomous machines are considered as a game changing technology that could revolutionize precision agriculture (PA) and facilitate the 'fourth agricultural revolution' often labelled ‘Agriculture 4.0’ (Daum, 2021; Klerkx & Rose, 2020; Lowenberg-DeBoer et al., 2021a, 2021b). Owing to population and economic growth, agricultural labour scarcity, technological advancement, increasing requirements of operational efficiency and productivity, and mitigating environmental footprint, autonomous machines are suggested as a sustainable intensification solution (Duckett et al., 2018; Guevara et al., 2020; Santos & Kienzle, 2020). Robotic systems for intensive livestock and for protected environments have been commercialized more rapidly than for arable cropping. Research on autonomous arable crop machines has mostly concentrated on the technical feasibility, not economics (Fountas et al., 2020; Shamshiri et al., 2018). Understanding the economic implications of autonomous machines is key to their long-term adoption. Economic feasibility plays a crucial role in attracting investment, guiding adoption decisions, and further understanding of environmental and social benefits (Grieve et al., 2019; Lowenberg-DeBoer et al., 2020).

Most production economic studies on autonomous machines prior to 2019 focused on horticultural crops and rarely on cereals using prototype testing and experimental data (Edan et al., 1992; Gaus et al., 2017; McCorkle et al., 2016; Pedersen et al., 2006, 2008, 2017; Sørensen et al., 2005). Lack of information on economic parameters and machinery specifications has been a bottleneck in economic feasibility assessment because autonomous machines are at an early stage of the development and commercialization processes (Lowenberg-DeBoer et al., 2021a, 2021b; Shockley et al., 2021). Most of the earlier economic studies used partial budgeting where only the changes in cost and revenue linked to automation of a single field operation were analysed omitting the economic consequences of farming systems changes (Lowenberg-DeBoer et al., 2020). To date, four studies have considered systems analysis of autonomous machines (Al-Amin et al., 2021; Lowenberg-DeBoer et al., 2021a, 2021b; Shockley et al., 2019; Sørensen et al., 2005).

Using a Linear Programming (LP) model with data from prototypes at the University of Kentucky, United States, Shockley et al. (2019) showed that relatively small autonomous machines are likely to have economic advantages for medium and small farms. The most comprehensive study so far was reported by Lowenberg-DeBoer et al., (2021a, 2021b). They assessed the economic feasibility of autonomous machines from seeding to harvesting operations using on-farm demonstration data and estimated equipment times based on methodology from the agricultural engineering textbook of Witney (1988). The study assumed 70% field efficiency from drilling to harvesting operations for both autonomous machines and conventional equipment sets with human operators. They showed that autonomous machines are technically and economically feasible for medium and small sized farms. The study concluded that autonomous machines diminished the pressure of “get big or get out”. The study hypothesized that in the context of the United Kingdom, autonomous machines would be economically feasible in small fields. Nonetheless, the study was unable to test the hypothesis because of field efficiency estimates by field size and shape were not available.

To help fill this knowledge gap, the objective of the study is to assess the economics of field size and shape for autonomous machines. Using the experience of the HFH demonstration project, the study developed algorithms to estimate equipment times (h/ha) and field efficiency (%) for different sized rectangular and non-rectangular fields. Historically, in the United Kingdom rectangular fields were considered as the most efficient, whereas non-rectangular fields were substantially less efficient to farm (Carslaw, 1930; Sturrock et al., 1977). Triangular fields were among the least efficient field shape because of the numerous short rounds. To analyse the economic scenarios, the study adopted and re-estimated the Hands Free Hectare-Linear Programming (HFH-LP) model (Lowenberg-DeBoer et al., 2021a, 2021b) by incorporating equipment times and field efficiency parameters estimated with field size and shape algorithms. The HFH-LP model replicates farm management and machinery selection decisions. It helps researchers understand choices that farmers would make if they had the alternative of using autonomous machine.

## Methods

### Field time and efficiency estimation subject to field size and shape

To date the production economics studies on autonomous machines did not consider field size and shape because of lack of data (Lowenberg-DeBoer et al., 2021a, 2021b; Shockley et al., 2019; Sørensen et al., 2005). Over time, the performance of arable field machinery has received growing attention for farm management and the ability to model field times has accelerated through the development of the technology and modelling approaches (Bochtis et al., 2010; Sørensen, 2003; Sørensen & Nielsen, 2005). Nonetheless, existing studies on arable crop machinery performance lack information of equipment times (h/ha) and field efficiency (%) subject to field size and shape.

Even though logistics software is well developed in trucking and other transportation sectors (Software Advice, 2021), there is no readily available commercial software in the United Kingdom to estimate equipment times and field efficiency encompassing field and machine heterogeneity. In the farm equipment path planning research literature, field times were sometimes generated as a by-product (Hameed, 2014; Jensen et al., 2012; Oksanen & Visala, 2007; Spekken & de Bruin, 2013). The agri-tech economic studies often rely on the general estimates of agricultural engineering textbooks like Hunt (2001) and Witney (1988). In conventional mechanization and PA literature, few studies estimated field efficiency, but prior studies treated the headlands of the field as non-productive areas, excluded overlap percentage, amalgamated productive field times (i.e., field passes, headlands turning, and headlands passes) and non-productive field times (i.e., replenish inputs, refuelling, and blockages), and ignored the headland turning patterns.

Studies suggested that future research should separately calculate the headlands turning time, and stoppages time because productive times and non-productive times play a significant role in field efficiency estimation. Keeping these points in consideration, the study developed field time approximation algorithms by field size and shape for 28 kW, 112 kW and 221 kW conventional equipment sets with human operators, and for the HFH sized 28 kW autonomous equipment set. The combine harvesters were assumed to have head widths of 2 m, 4.5 m and 7.5 m respectively. Using the experience of the HFH demonstration project, the algorithms addressed the research gaps identified from the prior studies. The study estimated field efficiency as the ratio of theoretical field time based on machine design specifications like the estimates of theoretical field time to its actual field productivity as follows:

1 $$ E_{f} = \left[ {T_{T} /\left( {T_{obs} + T_{h} + T_{sf} } \right)} \right] \times 100 $$

where, ${E}_{f}$ is the field efficiency, *T*_*T*_ is the theoretical field time, ${T}_{obs}$ is the total observed time in the interior field and passes, ${T}_{h}$ is the total headland round time, and ${T}_{sf}$ total stoppage time “within” in the field.

Based on user input of equipment and field measurements, the first step was to calculate field area, number of headlands rounds and other values that were used repeatedly throughout the algorithm. Secondly, headland area and field times were calculated. Afterwards, observed times in the interior field and passes were estimated. Fourthly, the algorithms estimated non-productive times. Fifthly, total field operation times were calculated. The theoretical field times were estimated based on the machine design specifications. For details of the estimation processes of the algorithms see the technical note in the Supplementary Material (i.e., STEXTT Supplementary Text).

**Fig. 1:**
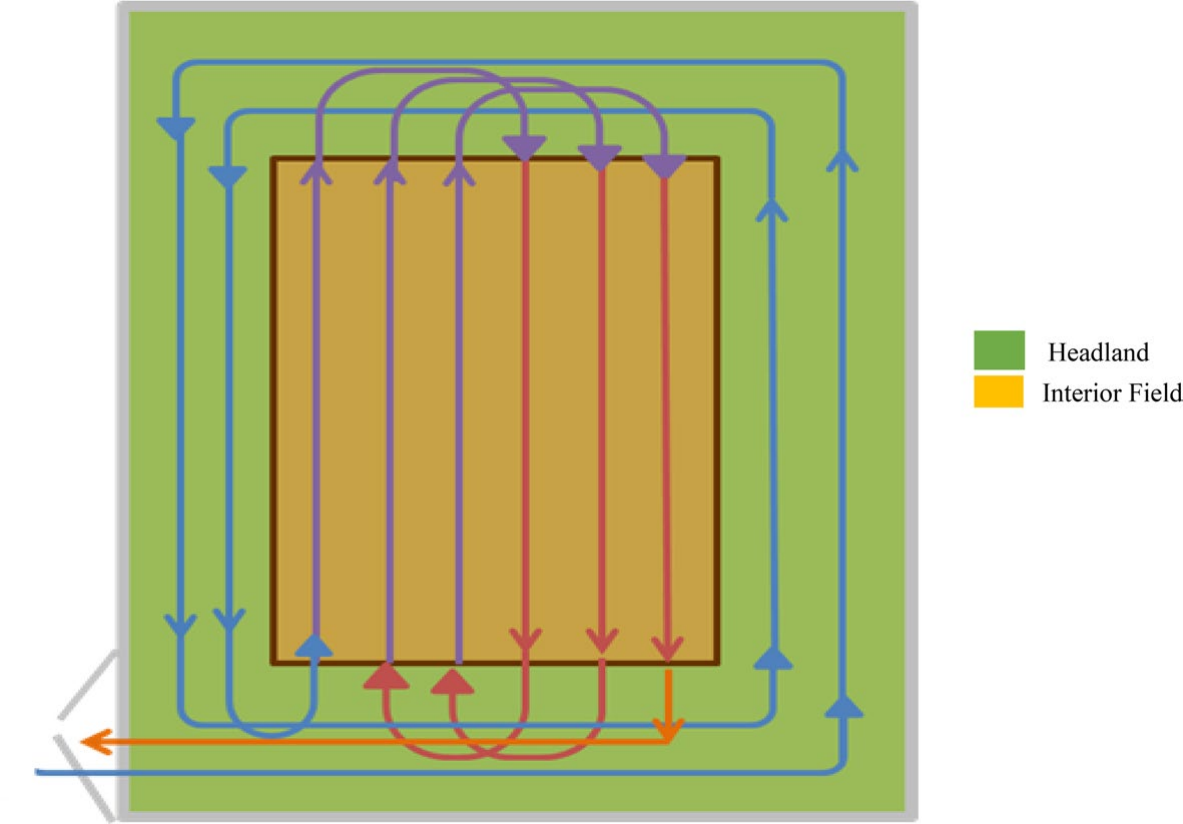
Typical field path for rectangular fields considered in the study based on the HFH demonstration project experience

The algorithms were calibrated for 1 ha, 10 ha, 20 ha, 50 ha, 75 ha, and 100 ha rectangular fields considering the typical farm field sizes of the United Kingdom that were assumed to follow the field path of Fig. 1. To illustrate the impact of field size on technical efficiency, estimates were made for rectangular fields with the length ten times the width of the field, up to one kilometre length. Rectangular field algorithms are detailed in the algorithms spreadsheet in the Supplementary Material (i.e., SM1 Rectangular Field Algorithms).

**Fig. 2:**
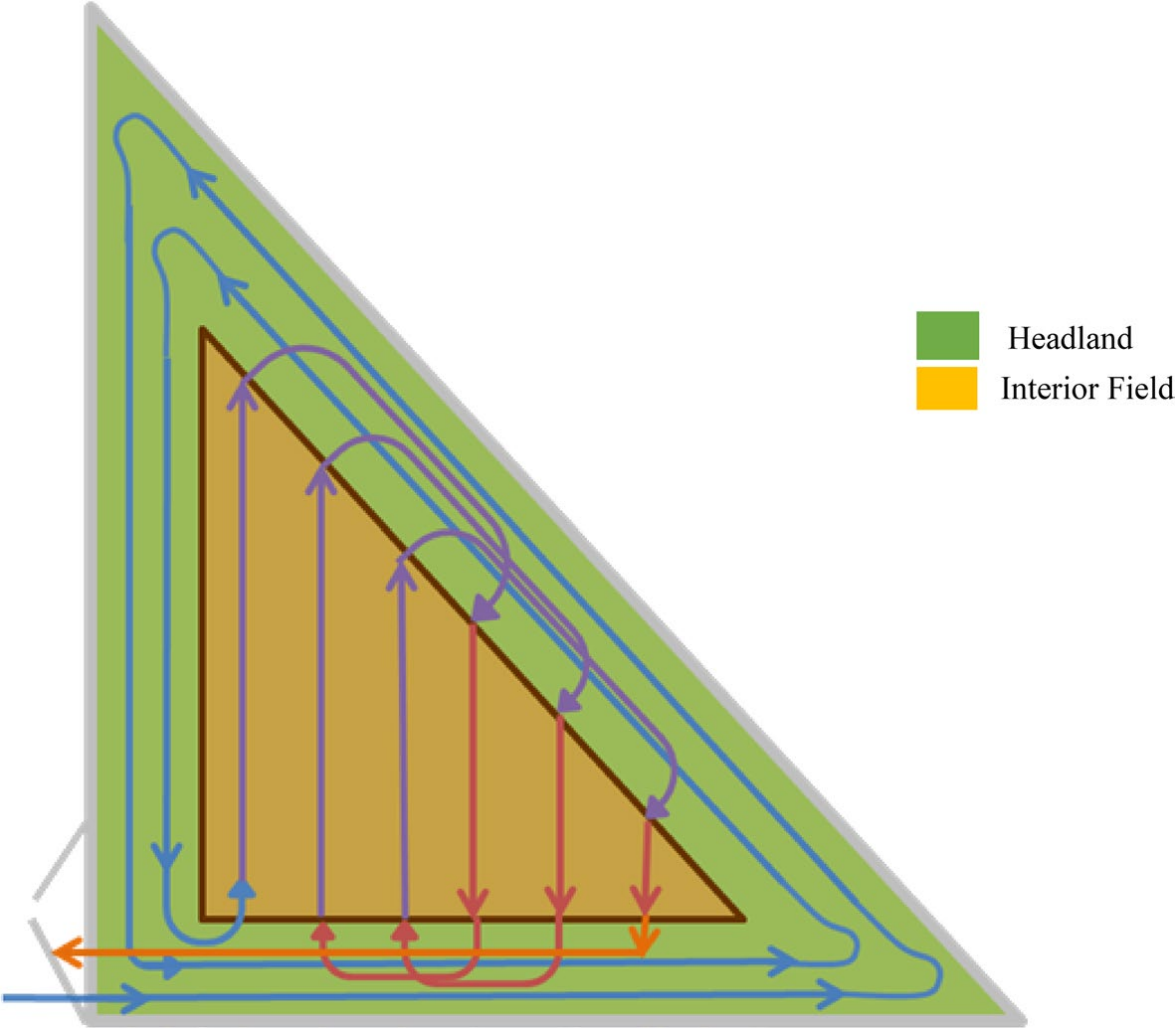
Typical field path for non-rectangular (i.e., right-angled triangular) fields considered in the study based on the HFH demonstration project experience

Similarly, non-rectangular fields algorithms were tested for 1 ha, 10 ha, 20 ha, and 25 ha sized right-angled triangular fields assuming the height equalling twice the base up to a height of one kilometer. The equipment sets were assumed to follow the typical field path given in Fig. 2. The non-rectangular fields algorithms were estimated with the same equipment sets (for details of the right-angled triangular field algorithms see spreadsheet in the Supplementary Material i.e., SM2 Non-Rectangular Field Algorithms).

The study assumed that the equipment enters the field from the lower left corner and completes the headlands first for all field operations (i.e., drilling, spraying, and harvesting). Afterwards, the machine makes a “flat turn” to start the interior passes. Subsequently, follows the “flat turn” to complete the interior headland turns. Finally, the study assumed that the equipment ends on the entry side of the fields as shown in Figs. 1 and 2.

### Modelling the economics of field size and shape

To understand the whole farm effects of field size and shape with different types of farm equipment, the study adopted and re-estimated the Hands Free Hectare-Linear Programming (HFH-LP) model. The HFH-LP model is a decision-making tool which assesses the economics of autonomous machines compared to conventional equipment sets with human operators. Consistent with typical neoclassical microeconomic farm theory, the objective function of the HFH-LP model was to maximize gross margin (i.e., return over variable costs) subject to primary farm resource constraints in the short-run. In the subsequent stages, using the outcome of the HFH-LP model, the study examined net return to operator labour, management and risk taking and evaluated the wheat cost of production to explore the cost economies (i.e., economies of size) (Debertin, 2012; Duffy, 2009; Hallam, 2017; Miller et al., 1981). The HFH-LP model is a one-year “steady state” model for arable grain-oil-seed farm, where the model assumed a monthly time step from January to December. It is steady state in the sense that it is assumed that solutions would be repeated annually long term. The concept of “steady state” was carried over from the Orinoquia model (Fontanilla-Díaz et al., 2021) which used the same software. Following Boehlje and Eidman (1984), the HFH-LP deterministic economic model can be expressed as:

The objective function:

2 $$Max \pi =\sum_{j=1}^{n}{c}_{j}{X}_{j}$$

Subject to:

3 $$\sum_{j=1}^{n}{a}_{ij}{X}_{j} \le {b}_{i} for i=1, \dots \dots , m;$$

4 $${X}_{j}\ge 0 for j=1, \dots \dots , n;$$

where, π is the gross margin, ${X}_{j}$ is the level of *j*th production activities, ${c}_{j}$ is the gross margin per unit over fix farm resources (${b}_{i}$) for the *j*th production activities, ${a}_{ij}$ is the amount of *i*th resource required per unit of *j*th activities, ${b}_{i}$ is the amount of available *i*th resource.

The HFH-LP model encompassed limiting constraints i.e., land, human labour, equipment times (i.e., tractor use time for drilling and spraying, and combine use time for harvesting), working capital and cashflow. The equipment scenarios encompassed four farm sizes: 66 ha, 159 ha, 284 ha and 500 ha farms, but did not model field size or shape. This study re-estimated the labour use, tractor use and combine use times for larger fields (10 ha) or smaller fields (1 ha), that were either rectangular or non-rectangular (i.e., right-angled triangular). The assumptions regarding variable costs, crop yields, and land use were same as Lowenberg-DeBoer et al., (2021b). The crop variable costs were the same across scenarios, but machinery costs differed. Details of the linear programming (LP) coefficients including machinery investment and operating costs are available from the supplementary materials of Lowenberg-DeBoer et al., (2021b). The 10 ha field size was selected for the large fields, because the field efficiency algorithm estimates showed that over 10 ha, field efficiency does not vary much by field size. A 1 ha field size was selected to represent small fields, because relatively few fields in the United Kingdom are smaller than 1 ha. The rectangular shape was selected as the shape usually considered most efficient for mechanized farming, and the triangular as the field shape that is among the least efficient (Carslaw, 1930).

The time window is crucial because agricultural operations are sensitive to weather conditions and crop activities. In literature the probability of good field days is considered as primary mechanism to model risk-aversion. The PC/LP model used good field days available in the 17th worst year out of 20 (McCarl et al., 1977) that is 85% of the time. Following Agro Business Consultants (2018) the study assumed that number of good field days available was in 4 years out of 5 years that is 80% of times. Similar to the original HFH-LP model, the conventional machines assumed that field operations of drilling, spraying and harvesting were conducted during day time that is on an average 10 h/day. The autonomous machines assumed that tractor for drilling and spraying was operated for 22 h/day (2 h for repair, maintenance and refuelling) while autonomous combine operated for 10 h/day limited for night dew. The LP models of the study were coded using the General Algebraic Modelling System (GAMS) (https://www.gams.com/). Details of other associated assumptions and the programming code is available at the supplementary materials of Lowenberg-DeBoer et al., (2021b).

### Case study and data sources

Because the Hands Free Hectare (HFH) was a demonstration project, it was difficult to separate on-field stops and down time while the engineers tinkered from those stoppages that would have occurred in normal field operations. Consequently, the model parameters were based on published machine specifications and farm budget information, and guided by the qualitative experience of the HFH project demonstrated at Harper Adams University, Newport, Shropshire, United Kingdom (Hands Free Hectare (HFH), 2021). The Lowenberg-DeBoer et al., (2021b) HFH-LP model represented the arable grain-oil-seed farm in the West Midlands of the United Kingdom, this study re-estimated field times to reflect the range of field sizes and shapes often found in Britain. To calibrate the HFH-LP model, the study used parameters from different sources. The information about commodity produced and the costs estimates were from the Agricultural Budgeting and Costing Book (Agro Business Consultants, 2018) and the Nix Pocketbook (Redman, 2018). To facilitate comparability with the Lowenberg-DeBoer et al., (2021b) results, 2018 input and output price levels were retained. Prices were converted following daily average exchange rate of 2018 from Great British Pounds (GBP) to Euro (€) of €1.1305 (Bank of England, 2018). Details of the machine inventory, costs of machines, hardware and software, crop rotations and key baseline assumptions are available at Lowenberg-DeBoer et al., (2021a, 2021b). Field operation timing was adopted from Finch et al. (2014) and Outsider’s Guide (1999).

Equipment timeliness (i.e., HFH 28 kW conventional equipment set with human operator and autonomous machine, 112 kW and 221 kW conventional equipment sets with human operators) were estimated through the developed algorithms, where the equipment and field specifications were collected from HFH demonstration experience (https://www.handsfree.farm/) (Hands Free Hectare (HFH), 2021), conventional machine specifications from John Deere (https://www.deere.co.uk/en/index.html) (John Deere, 2022), Arslan et al. (2014) and Lowenberg-DeBoer et al., (2021b). For more details of the technical parameters used and data sources see the Supplementary Materials (i.e., STEXTT Supplementary Text, SM1 Rectangular Field Algorithms and SM2 Non-Rectangular Field Algorithms).

## Results

### Field efficiency and times: rectangular fields

**Fig. 3:**
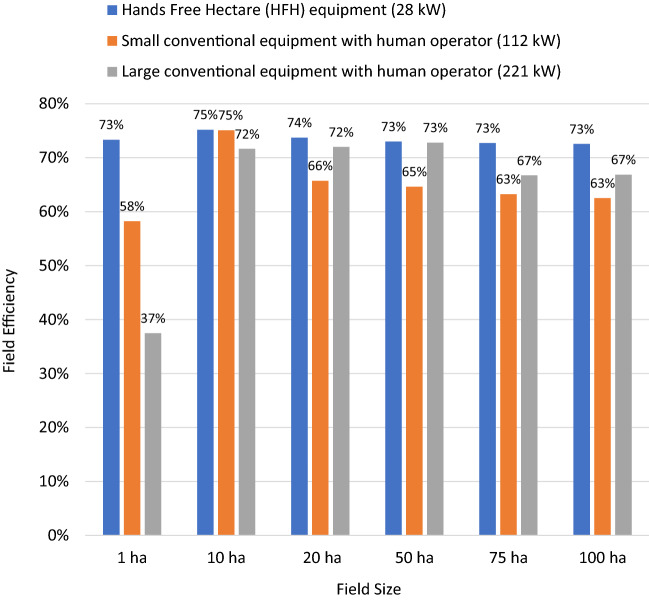
Estimated (weighted average) whole farm field efficiency of HFH equipment (i.e., 28 kW conventional equipment with human operator and autonomous machine), large conventional and small conventional machines with human operators in different sized rectangular fields

The study evaluated the technical feasibility of the HFH 28 kW conventional equipment with human operator and autonomous machines, and 112 kW and 221 kW conventional equipment sets with human operators for all field operations including direct drilling, five spray applications and harvesting operation. The spray application included pre-drill burn down, two nitrogen top dressing and fungicide applications, late season fungicide and pre-harvest desiccant. The human and equipment times were re-estimated subject to field size and shape scenarios. Results show that average whole farm field efficiency for 112 kW and 221 kW equipment sets differed substantially between 1 and 10 ha rectangular fields, whereas for rectangular fields a given equipment set the field efficiency was almost the same for 10 ha to 100 ha fields (Fig. 3). The whole farm field efficiency of HFH equipment sets was relatively high irrespective of different sized rectangular fields, but efficiency for 112 kW and 221 kW conventional equipment sets with human operators dropped for small 1 ha fields. Beyond 10 ha, the field efficiency for a given equipment set was similar for all rectangular field sizes (i.e., 20 ha, 50 ha, 75 ha, and 100 ha).

Operation specific equipment times (h/ha) and field efficiency (%) results of the rectangular fields show that equipment times for drilling and harvesting operations were longer for small 1 ha fields operated with equipment of all sizes and types, but field sizes had least impact for the HFH equipment sets (Table 1). The higher time for small 1 ha fields was largely due to the fact that the full width of the larger equipment could not be used effectively in the smaller fields.

**Table 1:** Equipment times of the machinery sets for rectangular fields of 1 ha and 10 ha

Equipment	Width of the implement (m)^b^	Overlap percentage (%)^b^	Field speed (km/h)^b^	Field efficiency (%)^c^	Field times (h/ha)
1 ha rectangular field					
HFH equipment set (28 kW)^a^					
Drill	1.5	10	3.25	81	2.81
Sprayer	7	10	5	71	0.45
Combine	2	10	3.25	78	2.19
Larger conventional set (221 kW)					
Drill	6	10	5	46	0.81
Sprayer	36	10	10	34	0.09
Combine	7.5	10	3	44	1.12
Small conventional set (112 kW)					
Drill	3	10	5	69	1.07
Sprayer	24	10	10	56	0.08
Combine	4.5	10	3	59	1.39
10 ha rectangular field					
HFH equipment set (28 kW)^a^					
Drill	1.5	10	3.25	89	2.56
Sprayer	7	10	5	69	0.46
Combine	2	10	3.25	91	1.88
Larger conventional set (221 kW)					
Drill	6	10	5	87	0.43
Sprayer	36	10	10	65	0.05
Combine	7.5	10	3	87	0.57
Small conventional set (112 kW)					
Drill	3	10	5	94	0.79
Sprayer	24	10	10	70	0.07
Combine	4.5	10	3	83	0.99

^a^HFH equipment sets are 28 kW conventional machine with human operator and 28 kW autonomous machine

^b^The machine specifications and overlap assumptions were collected from the HFH experience and Lowenberg-DeBoer et al., (2021b)

^c^The authors developed algorithms to estimate the field efficiency of rectangular fields (for details of the estimation procedures and algorithms see the technical note and excel spreadsheet in the supplementary materials i.e., STEXTT Supplementary Text and SM1 Rectangular Field Algorithms)

### Field efficiency and times: non-rectangular fields

**Fig. 4:**
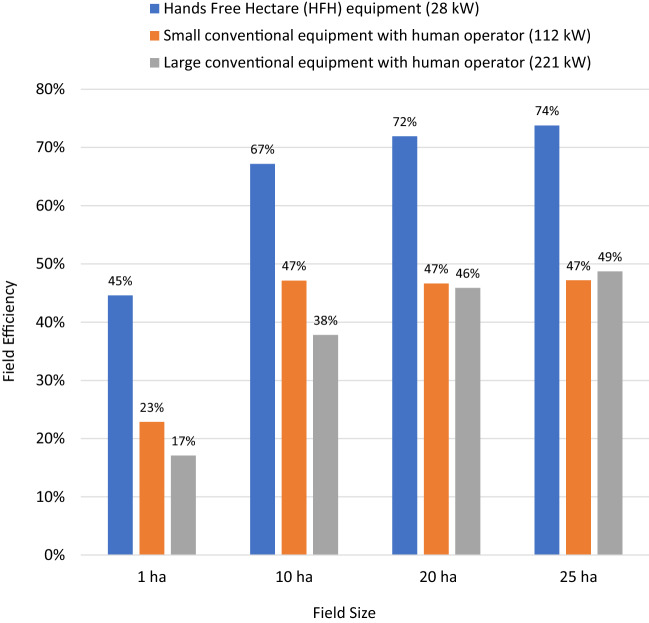
Estimated (weighted average) whole farm field efficiency of HFH equipment (i.e., 28 kW conventional equipment with human operator and autonomous machine), large conventional and small conventional machines with human operators in different sized non-rectangular fields

The average whole farm field efficiency for non-rectangular (i.e., right-angled triangular) fields differed substantially between 1 and 10 ha fields, but for a given equipment set the average whole farm field efficiency was almost the same for 20 ha and 25 ha fields (Fig. 4). The technical feasibility (i.e., field times and field efficiency) results show that HFH 28 kW equipment sets were more efficient than larger equipment for all sized non-rectangular fields even in small 1 ha fields.

The equipment times were longer for all operations in small 1 ha non-rectangular fields equipped with equipment of all sizes and types, but field sizes had least impact for the HFH equipment sets (Table 2). The higher time for small 1 ha fields was largely due to the fact that the full width of the larger equipment could not be used effectively in the smaller fields. Drilling operations required the highest equipment times and subsequently followed by harvesting and spraying in case of HFH 28 kW equipment sets, whereas for conventional equipment sets with human operators (i.e., 221 kW and 112 kW) irrespective of field sizes, harvesting consumed more time, followed by drilling and spraying. The non-rectangular 1 ha and 10 ha fields had comparatively lower field efficiency and required longer equipment times than rectangular fields with the same area. Small 1 ha non-rectangular fields required more time for field operations than the rectangular fields due to the varying interior length of the passes and higher interior headlands turning time for a given field area. The comparatively lower times for spraying compared to drilling and harvesting operations was associated to the field and equipment specifications of the sprayer because the sprayers were the widest implement. This is also resulted in the lower field efficiency for spraying small fields (detailed estimation of field times for non-rectangular fields are available in the Supplementary Material i.e., SM2 Non-Rectangular Field Algorithms).

**Table 2:** Equipment times of the machinery sets for non-rectangular fields of 1 ha and 10 ha

Equipment	Width of the implement (m)^b^	Overlap percentage (%)^b^	Field speed (km/h)^b^	Field efficiency (%)^c^	Field times (h/ha)
1 ha non-rectangular field					
HFH equipment set (28 kW)^a^					
Drill	1.5	10	3.25	47	4.85
Sprayer	7	10	5	44	0.72
Combine	2	10	3.25	45	3.80
Larger conventional set (221 kW)					
Drill	6	10	5	20	1.85
Sprayer	36	10	10	16	0.19
Combine	7.5	10	3	19	2.60
Small conventional set (112 kW)					
Drill	3	10	5	27	2.74
Sprayer	24	10	10	22	0.21
Combine	4.5	10	3	24	3.43
10 ha non-rectangular field					
HFH equipment set (28 kW)^a^					
Drill	1.5	10	3.25	70	3.26
Sprayer	7	10	5	66	0.48
Combine	2	10	3.25	71	2.41
Larger conventional set (221 kW)					
Drill	6	10	5	43	0.86
Sprayer	36	10	10	36	0.09
Combine	7.5	10	3	41	1.20
Small conventional set (112 kW)					
Drill	3	10	5	54	1.37
Sprayer	24	10	10	46	0.10
Combine	4.5	10	3	48	1.71

^a^HFH equipment sets are 28 kW conventional machine with human operator and 28 kW autonomous machine

^b^The machine specifications and overlap assumptions were collected from the HFH experience and Lowenberg-DeBoer et al., (2021b)

^c^The authors developed algorithms to estimate the field efficiency of a right-angled triangular fields (for details of the estimation procedures and algorithms see the technical note and excel spreadsheet in the supplementary material i.e., STEXTT Supplementary Text and SM2 Non-Rectangular Field Algorithms)

### Economics of rectangular fields

The HFH-LP solutions for the farm size, field size and equipment set scenarios for rectangular fields are presented in Table 3. For a given farm size gross margins differed only slightly by equipment set and field size. The small differences by field size are due to small changes in cropping plan and the need to hire more labour with smaller fields. Only variable costs are deducted in calculating gross margin, so the costs of different equipment sets are not reflected in that measure of profit. The identical gross margin for 66 ha farms with 1 ha and 10 ha sized rectangular fields is because the smallest farms operated with four equipment scenarios did not face any operator and labour time constraints even with 1 ha fields and consequently planted, maintained and harvested the wheat-oilseed rape (OSR) rotation at optimal times regardless of field size.

**Table 3:** HFH-LP outcomes on the economics of technology choice subject to different sized rectangular fields

Scenario^a^	Arable area (ha)^b^	Field size (ha)	Labour hired in the farm (person-days per annum)	Operator time required in the farm (person-days per annum)	Whole farm gross margin (€ per annum)	Return to operator labour, management and risk taking (€ per annum)	Wheat cost of production with allocated operator labour (€ per ton)
Conv. 28 kW	59.4	10	0	66	53188	17916	181
Conv. 28 kW	59.4	1	0	72	53188	17916	185
Conv. 28 kW^2^	143.1	10	39	119	124670	43937	167
Conv. 28 kW^2^	143.1	1	55	118	123314	42580	168
Conv. 28 kW^3^	255.6	10	138	145	216729	78453	156
Conv. 28 kW^3^	255.6	1	165	144	214348	76072	157
Conv. 28 kW^4^	450.0	10	326	172	374197	144028	147
Conv. 28 kW^4^	450.0	1	371	172	369151	138983	148
Autonomous 28 kW	59.4	10	0	19	53188	13906	154
Autonomous 28 kW	59.4	1	0	22	53188	13906	156
Autonomous 28 kW	143.1	10	0	46	128134	53747	138
Autonomous 28 kW	143.1	1	0	53	1281352	53747	140
Autonomous 28 kW^2^	255.6	10	22	60	226922	90983	137
Autonomous 28 kW^2^	255.6	1	33	61	225961	90022	137
Autonomous 28 kW^3^	450.0	10	72	73	396560	164718	132
Autonomous 28 kW^3^	450.0	1	92	74	394869	163026	132
Conv. 112 kW	59.4	10	0	23	53188	− 29394	236
Conv. 112 kW	59.4	1	0	32	53188	− 29394	243
Conv. 112 kW	143.1	10	0	56	128134	10448	174
Conv. 112 kW	143.1	1	6	71	127629	9943	180
Conv. 112 kW	255.6	10	19	82	227171	62300	153
Conv. 112 kW	255.6	1	45	92	224916	60045	155
Conv. 112 kW^2^	450.0	10	78	100	396082	92008	153
Conv. 112 kW^2^	450.0	1	139	102	390675	86602	154
Conv. 221 kW	59.4	10	0	14	53188	− 80235	323
Conv. 221 kW	59.4	1	0	26	53188	− 80235	333
Conv. 221 kW	143.1	10	0	33	128134	− 40394	206
Conv. 221 kW	143.1	1	0	63	128134	− 40394	216
Conv. 221 kW	255.6	10	0	59	228869	13157	170
Conv. 221 kW	255.6	1	28	86	226438	10725	175
Conv. 221 kW	450.0	10	20	84	401162	103917	148
Conv. 221 kW^2^	450.0	1	95	105	394593	− 11163	179

^a^The superscript with equipment specification under scenario indicates the number of equipment sets

^b^Based on the experience of HFH demonstration project, the study assumed that the arable crop farm was 90% tillable, where remaining 10% were occupied for ecologically focused area such as, lanes, hedgerows, drainage ditches, farmstead, etc.

For a given farm size, the net return to operator labour, management and risk taking vary more by equipment set, than by field size because of the differences in equipment costs. Except for the smallest farm, the net returns at a given farm size are highest for the autonomous machine scenarios. Net return for the smallest farm was higher when using a 28 kW conventional equipment set with human operator than with autonomous machines mainly because of the cost of retrofitting the equipment set for autonomy, but it is important to note that the conventional scenario required over 3 times more operator time than the autonomous machines. The higher return to operator labour, management and risk taking for the 66 ha farm using conventional equipment is because that measure of profit is a residual after cash costs, but does not deduct for operator compensation. The conventional solution maximizes net return for the smallest farm only if operator labour has a very low opportunity cost.

**Fig. 5:**
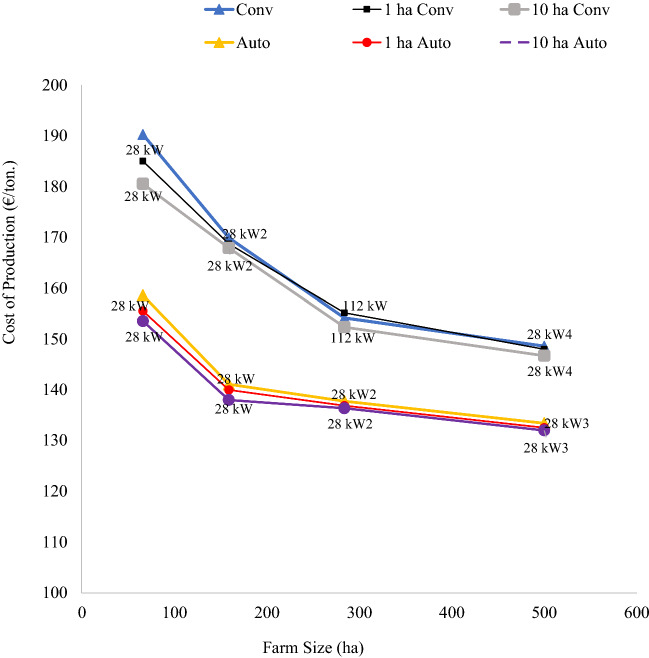
Wheat unit cost of production in euro per ton for farms with rectangular fields of different sized farms. The labels on the data points for 1 ha and 10 ha fields are the size of the tractor used and the number of equipment sets. The curves without labels are the baseline analysis which was done without field size and shape modelling

The debate about economies of size (i.e., whether increasing economies of size, decreasing economies of size known as diseconomies of size, or constant economies of size) has been carried on largely in terms of cost curves in agricultural production economics (Debertin, 2012; Duffy, 2009; Hallam, 2017). Building on this literature, the study estimated the wheat production cost of mechanized farms with different sized and shaped fields equipped with autonomous machines and conventional equipment sets with human operators. The wheat production cost curves were estimated based on the most profitable farm plans that cultivated all available land with minimum unit production cost for their size. The study hypothesized that irrespective of field size and shape, autonomous machines would have lower wheat production cost and reduced economies of size compared to conventional equipment sets with human operators. The wheat cost of production curves for rectangular fields were similar to those estimated without considering field size, with conventional equipment showing higher production cost. The cost curves for autonomous machines with both field sizes lie below the curves for conventional equipment sets with human operators (Fig. 5). Autonomous machine wheat production cost scenarios indicating that irrespective of field sizes autonomous systems had lower cost of production and reduced economies of size compared to farms operated with conventional equipment sets. The autonomous machines cost advantage was mainly due to lower labour and machine costs. The reduced economies of size for autonomous machines’ cost curves (i.e., 1 ha curve represented by smaller circular marker curve and 10 ha curve with bigger circular marker) can be seen in costs levelling off with a relatively flat bottom at smaller scale compared to the cost curves with conventional equipment sets with human operators. As farm size increases, cost curves for autonomous machines showed similar cost of production irrespective of field sizes. The additional cost curve (i.e., triangular marker curve) above 1 ha and 10 ha rectangular fields represents the wheat production cost curve for autonomous machines without field size consideration estimated by Lowenberg-DeBoer et al., (2021b) taken as base category for comparison.

The wheat cost scenarios by equipment set shows that compared to conventional equipment sets, the autonomous machines reduced wheat cost of production by €15/ton to €29/ton for 1 ha rectangular fields. The wheat cost of production curves with conventional equipment sets (28 kW, 112 kW, and 221 kW) reveal that farms with 1 ha and 10 ha rectangular fields had substantial effect on per unit wheat cost of production. The minimal cost difference between 1 and 10 ha sized fields wheat cost of production curves was associated with the lower differences of field times and field efficiency for rectangular fields.

## Sensitivity tests: rectangular fields

Because agricultural labour is scarce and difficult to hire in the United Kingdom, some of the HFH LP conventional farm solutions may be difficult to implement and consequently the cost curves may not be realistic. For example, for the 500 ha farm the cost curves for conventional machines in both field size scenarios reveal that minimum wheat cost of production was achieved with four 28 kW equipment sets. For that 500 ha conventional farm the 10 ha solution required 326 days of hired labour and the 1 ha solution required 371 days, compared to 72 days and 92 days respectively for the autonomous farm. To test the sensitivity of solutions to the cost of labour, the model was rerun with the wage rate doubled (i.e., €11.02 × 2 × 8 = €176/day). With the higher wage rate, the minimum cost for the 500 ha farm with 10 ha fields was achieved at €148/ton with a 221 kW equipment set, but for 1 ha fields minimum cost at €156/ton was still achieved with four units of 28 kW equipment set as earlier. Additional sensitivity tests with triple wage rate (i.e., €11.02 × 3 × 8 = €264/day) show that for 500 ha farm with 10 ha fields minimum costs (i.e., €149/ton) achieved as earlier with a 221 kW equipment set, whilst for 500 ha farm with 1 ha fields minimum costs (i.e., €160/ton) achieved with two units of 112 kW equipment set. With higher wage rates the shape of the cost curves for the conventional farms indicated less cost advantage for larger farms irrespective of field sizes (for details see SFigs. 1 and 2 in the Supplementary Material i.e., SFs Sensitivity Tests Figure).

Further sensitivity scenarios considered hired labour constrained at 50 person days per month with baseline wage rate (€11.02 × 8 = €88/day), where the optimum solution with minimum cost (€156/ton) for larger 500 ha farm with 1 ha fields, were achieved with two units of 112 kW equipment set and for 10 ha fields minimum costs at (€148/ton) with a 221 kW equipment set (for details see SFig. 3). Consequently, the use of multiple conventional 28 kW equipment sets with human operators were not feasible solutions with higher wage rates and less labour availability. Moreover, the sensitivity tests with increasing wage rates and reduced labour availability scenarios show a more distinct gap between cost curves for farms with 1 ha and 10 ha fields, because 1 ha fields required substantially more labour.

## Economics of non-rectangular fields

For non-rectangular fields, the machinery and field size scenarios show that gross margin and net return to operator labour, management and risk-taking patterns were similar to those of the rectangular fields (Table 4). Net returns differed more by equipment set than field size, but the field size effect was more pronounced than for rectangular fields. The identical gross margin for 66 ha farms with 10 ha sized non-rectangular fields is because the smallest farms did not face any operator and labour time constraints, therefore they planted, maintained and harvested the wheat-OSR rotation at optimal times. On the contrary, gross margins for 66 ha farm with 1 ha non-rectangular fields were higher for autonomous machines and larger conventional equipment compared to 28 kW and 112 kW conventional equipment sets because these two conventional sets faced operator time constraints and required more hired labour for farm operations.

**Table 4:** HFH-LP outcomes on the economics of technology choice subject to different sized non-rectangular fields

Scenario^a^	Arable area (ha)^b^	Field size (ha)	Labour hired in the farm (person-days per annum)	Operator time required in the farm (person-days per annum)	Whole farm gross margin (€ per annum)	Return to operator labour, management and risk taking (€ per annum)	Wheat cost of production with allocated operator labour (€ per ton)
Conv. 28 kW	59.4	10	0	80	53188	17916	191
Conv. 28 kW	59.4	1	16	106	51796	16525	213
Conv. 28 kW^2^	143.1	10	71	121	121863	41129	171
Conv. 28 kW^2^	143.1	1	144	149	111684	30950	190
Conv. 28 kW^3^	255.6	10	195	147	211630	73354	158
Conv. 28 kW^4^	255.6	1	355	169	197539	48904	173
Conv. 28 kW^4^	450.0	10	415	188	341261	111092	159
Conv. 28 kW^7c^	450.0	1	743	180	337415	76172	164
Autonomous 28 kW	59.4	10	0	24	53188	13906	157
Autonomous 28 kW	59.4	1	0	38	53188	13906	167
Autonomous 28 kW	143.1	10	3	55	127832	53446	141
Autonomous 28 kW^2^	143.1	1	31	60	125421	36666	155
Autonomous 28 kW^2^	255.6	10	41	63	225279	89340	138
Autonomous 28 kW^3^	255.6	1	90	72	220972	70664	147
Autonomous 28 kW^3^	450.0	10	105	77	393668	161827	133
Autonomous 28 kW^4^	450.0	1	191	94	386084	139875	140
Conv. 112 kW	59.4	10	0	40	53188	− 29394	249
Conv. 112 kW	59.4	1	3	76	52612	− 29970	278
Conv. 112 kW	143.1	10	17	78	126621	8934	182
Conv. 112 kW^2^	143.1	1	93	99	119892	− 55463	239
Conv. 112 kW	255.6	10	74	96	222323	57452	157
Conv. 112 kW^2^	255.6	1	231	112	208461	− 14078	190
Conv. 112 kW^2^	450.0	10	193	107	385913	81840	156
Conv. 112 kW^4c^	450.0	1	470	135	361510	− 57900	192
Conv. 221 kW	59.4	10	0	28	53188	− 80235	335
Conv. 221 kW	59.4	1	0	60	53188	− 80235	358
Conv. 221 kW	143.1	10	0	67	128134	− 40394	217
Conv. 221 kW	143.1	1	49	96	123812	− 44716	228
Conv. 221 kW	255.6	10	33	87	225987	10275	175
Conv. 221 kW^2^	255.6	1	149	109	215702	− 108520	231
Conv. 221 kW^2^	450.0	10	106	105	393552	− 12205	179
Conv. 221 kW^3c^	450.0	1	325	130	374258	− 140009	213

^a^The superscript with equipment specification under scenario indicates the number of equipment sets

^b^Based on the experience of HFH demonstration project, the study assumed that the arable crop farm was 90% tillable, where remaining 10% were occupied for ecologically focused area such as, lanes, hedgerows, drainage ditches, farmstead, etc.

^c^The study baseline scenarios assumed a maximum of 100 person-days/month of temporary labour available, but in the sensitivity testing that was raised to 200 person-days/month

Economic scenarios of non-rectangular fields incorporating fixed costs show that net returns to operator labour, management, and risk taking were higher for autonomous machines irrespective of field sizes, except for the smallest 66 ha farm equipped with 28 kW conventional machine with human operator. As with non-rectangular fields, this is because the autonomous machines required extra cost for retrofitting equipment for autonomy. The higher net return to operator labour, management and risk taking for the conventional 66 ha farm may be an illusion because of the higher labour requirement. For the 66 ha farm with 10 ha fields, no labour was hired in either conventional or autonomous scenarios, but the conventional farm required 3 times more operator labour time than the autonomous farm. For the 66 ha farm with 1 ha fields, the conventional farm required 3 times more operator labour, plus 16 days more hired labour. As discussed above in regard to the rectangular case, a small conventional 28 kW equipment set is not the sustainable solution given the growing labour scarcity in arable farming in the United Kingdom.

**Fig. 6:**
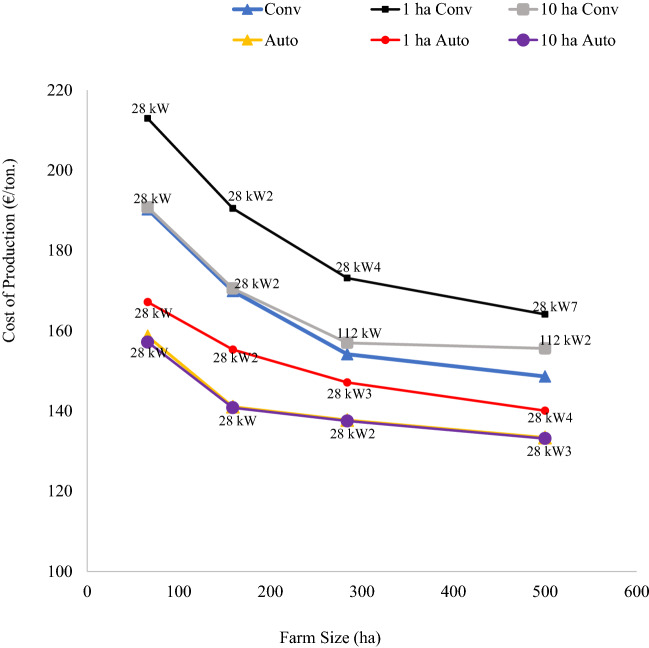
Wheat unit cost of production in euro per ton for farms with non-rectangular fields of different sized farms. The labels on the data points for 1 ha and 10 ha fields are the size of the tractor used and the number of equipment sets. The curves without labels are the baseline analysis which was done without field size and shape modelling

The wheat cost of production curves with non-rectangular fields shows that irrespective of field sizes, farms with autonomous machines had cost advantages (i.e., lower cost of production) and reduced economies of size compared to farms with conventional equipment sets with human operators (Fig. 6), but the field size effect is more evident than rectangular fields.

More specifically, the autonomous cost curves scenarios reveal that small 1 ha non-rectangular fields entailed higher wheat cost of production compared to 10 ha fields, which was associated with comparatively higher hired labour, operator time and equipment scenarios (i.e., number of equipment required). The equipment scenarios show that small non-rectangular fields required more autonomous equipment sets to optimally operate the same farm, except for the smallest farm. Likewise, for conventional equipment sets, small 1 ha fields had substantially higher wheat production costs compared to 10 ha fields. For larger 500 ha farms equipped with conventional sets, the minimum unit cost of production was achieved with seven-units of 28 kW equipment set for 1 ha fields, whereas 10 ha fields had minimum unit cost scenarios with two units of 112 kW equipment sets. The wheat cost scenarios by equipment set shows that autonomous machines reduced wheat cost of production by €24/ton to €46/ton in 1 ha non-rectangular fields, indicating that autonomous equipment has cost advantage (i.e., lower cost of production) and reduce economies of size compared to conventional equipment sets with human operators.

## Sensitivity tests: non-rectangular fields

The sensitivity tests with wage rate double and triple for mechanized non-rectangular fields reveal that all farms irrespective of field sizes were able to operate profitably with minimum wheat cost of production using the same equipment scenarios as with the baseline wage rate (i.e., €11.02 × 8 = €88/ton) equipment scenarios. However, with the increasing wage rates, the profitable farms had to incur more per unit production cost for all conventional and autonomous equipment sets. Small 1 ha fields operated with conventional equipment sets with human operators had to take more cost burden. For instance, 500 ha farms with small 1 ha fields had to incur €16/ton and €32/ton more costs with double and triple wage rates scenarios. Interestingly, even with double and triple wage rates, the autonomous machines had the lower cost of production and reduced economies of size compared to the conventional mechanized farms (for details see SFigs. 4 and 5 in the Supplementary Material i.e., SFs Sensitivity Tests Figure).

Further sensitivity test with reduced labour availability at 50 person days per months reveal that multiple conventional equipment scenarios with human operators were not an economically feasible solution. For example, with the base wage rate scenario, 500 ha farms with 10 ha fields achieved minimum costs at €156/ton operated with two units of 112 kW equipment sets with human operators, whilst in case of reduced labour availability, the minimum cost was achieved at €167/ton with the same equipment scenarios, indicating diseconomies of size. Moreover, the reduced labour availability made larger conventional mechanized farms plans (i.e., 284 ha and 500 ha) with small 1 ha fields non-economical and unrealistic because the existing conventional equipment sets with human operators (i.e., 28 kW, 112 kW and 221 kW) were unable to cultivate the optimum land with the available resources of the farms. The 500 ha farm with small 1 fields equipped with four units of autonomous machines were unable to operate the optimum land, that was 53 (450 − 397.41 = 52.59 ha), 1 ha fields were left unutilized with the available resources of the farms.

## Discussion

The economic implications of field size and shape, contributes to the cost economies literature as prior production economies studies did not include the economics of field size and shape for autonomous machinery. The present study filled the research gap with the findings that irrespective of field size and shape, autonomous machines had lower wheat production cost and reduced economies of size compared to conventional equipment sets with human operators.

Throughout the world agricultural labour is difficult to hire and wage rate is increasing. In addition, the Covid-19 pandemic sparked the labour scarcity. These real-world crises spurred the study to further investigate the sensitivity scenarios with increasing wage rates and reduced labour availability. Considering the context of the United Kingdom, the sensitivity scenarios of double and triple wage rates and reduced labour availability reveal that irrespective of field size and shape, multiple conventional equipment sets with human operators were not a good solution for small fields. Autonomous machines (i.e., autonomous swarm robotics) were an economically feasible alternative in the face of rising wage rates ensuring the lower cost of production and more competitiveness for medium and small farms. Under the reduced labour availability scenario, autonomous machines allowed available labour to farm more land with lower cost of wheat production than the conventional equipment scenario, but with small, non-rectangular fields even the autonomous machines faced binding labour constraints. This is primarily due to the continued need for some human supervision and for human operators on public roads [i.e., assumption of 10% human supervision time during field operations and 100% human operators driving in the public road for hauling grain from the field to farmstead or market during harvest based on the study of Lowenberg-DeBoer et al., (2021b)].

The results support the hypothesis of the study that autonomous machines offer the possibility of farming small fields profitably, implying the potentials of biodiversity enhancement and environmental performance of such small fields as a side effect (Fahrig et al., 2015; Firbank et al., 2008; Konvicka et al., 2016). The autonomous arable crop farms could support the United Kingdom’s agricultural transition plan for sustainable farming. The economic feasibility of small autonomous farms facilitates implementation of the UK government Environmental Land Management (ELM) Scheme focused on encouraging agriculture to provide environmental public goods including improved soil health, greater field biodiversity and carbon sequestration (DEFRA, 2020, 2021). Likewise, the study supports agri-environment schemes (AES) to encourage small fields for biodiversity in the European Union and elsewhere (Geppert et al., 2020).

The findings of the study also provide information to guide decision making by farmers, agribusinesses, technology developers, and policymakers. More specifically, the study guides “*farm size and shape policy*” generally associated with *“agricultural mechanization policy”* and *“biodiversity conservation policy”* of large (i.e., Brazil, Argentina, United States, Australia, and Mexico) and medium (i.e., United Kingdom and Europe) scale farming to develop policies considering environmental performance in arable farming. The profitability of autonomous farms with small fields irrespective of field size and shape, indicate that the rule of thumb of conventional mechanized agriculture (i.e., “get big or get out” and promoting “structural change of arable landscapes”) will be superseded with autonomous machines.

However, this study has some limitations in the development of algorithms and existing economic modelling scenarios. Because of data deficiencies, the algorithms assumed zero down time due to machine problems (e.g., seed tines blocked with crop residue, plugged sprayer nozzles, damp straw wrapping a combine harvester drum). Hands Free Hectare (HFH) was a demonstration project, so it was difficult to separate stops for research purposes and those that would have occurred on any farm. Future research could reinvestigate this assumption based on farm experience. In terms of technical and economic modelling scenarios, the study only considered four equipment sets (28 kW, 112 kW and 221 kW conventional equipment set with human operators and 28 kW autonomous machines retrofitted for autonomy); there may be other equipment sizes that may better fit the given circumstances, especially for small 1 ha rectangular and non-rectangular fields. The study assumed same field times and efficiency for 28 kW machines whether autonomous or human operated. In the future autonomous machines may be equipped with improved technology that reduce field times and increase efficiency beyond even the best human operator, but the conservative assumption for this study was that they were the same for the 28 kW machines whether conventional or autonomous. In addition to the large and medium scale farming, future research should consider the context of small-scale farming (i.e., most of Asia and Africa), with field sizes tiny, fragmented fields of less than 1 ha. Some observers have hypothesized that autonomous machines would be technically and economically feasible solution for labour scarcity problems on small farms, especially in peak production seasons (Al-Amin and Lowenberg-DeBoer, 2021; HLPE, 2013; Lowder et al., 2016).

This study focused tightly on the implications of field size and shape for the economics of autonomous machines in the United Kingdom. Because high and medium income countries and even in many cases the low income countries throughout the world face labour scarcity in agriculture (Lowenberg-Deboer, 2022; World Bank, 2021a, 2021b), the methodology could be adapted to estimate the economic implications of autonomous equipment in other cropping systems with different challenges. Future economic research could address other associated benefits of autonomous machines such as reduced fuel use or alternative renewable fuel use, potentials of mixed cropping like pixel, patch, strip, relay cropping and regenerative agriculture (Davies, 2022; Ditzler & Driessen, 2022; Hein, 2022; Ward et al., 2016). Even though, the technical and economic feasibility of autonomous machines in small, non-rectangular fields show potential for improving environmental management, future research should incorporate field inclusions, such as in field trees and wetlands. These inclusions may address field topography issues like grass waterways of Batte and Ehsani (2006) and/or encourage aboveground environmental diversification with intercropping and non-crop habitat such as flower strips, hedgerows and seminatural habitats within the field or around the field (Bellon-Maurel & Huyghe, 2017; Boeraeve et al., 2020; Tamburini et al., 2020). Similarly, the economic implications of soil compaction with low weight autonomous machines random trafficking fields could be compared to larger/heavier machines conventional or autonomous machines working in a controlled traffic setting (Berli et al., 2004; Keller & Or, 2022; Keller et al., 2019; Shockley et al., 2021).

## Conclusions

The study contributed to the cost economies literature with the findings that irrespective of field size and shape, autonomous machines had lower wheat production cost and reduced economies of size compared to conventional equipment sets with human operators. This study hypothesized that autonomous crop machines would make it possible to farm small, non-rectangular fields profitably, thereby preserving field biodiversity and other environmental benefits. To test the hypothesis, the study developed algorithms to estimate field efficiency (%) and equipment times (h/ha) for different sized rectangular and non-rectangular (i.e., right angled triangular) fields. Algorithm results show that the smallest equipment considered (i.e., HFH 28 kW conventional equipment set with human operator and retrofitted autonomous machines) required more time per hectare, but had higher field efficiency irrespective of field size and shape, compared to the conventional equipment sets with human operators (i.e., 221 kW and 112 kW). This was true for both rectangular and non-rectangular fields. Economic scenarios (i.e., return over variable costs and net return to operator labour, management, and risk taking) examined with mathematical programming (i.e., HFH-LP models) show that autonomous machines were a profitable solution for arable farms with small fields. The wheat production cost curves comparison reveal that autonomous machines reduced cost of production by €15/ton to €29/ton for farms with small 1 ha rectangular fields. For farms with 1 ha non-rectangular fields per unit wheat production cost was reduced by €24/ton to €46/ton. The ability of autonomous crop machines to profitably farm small, irregularly shaped fields, even with increasing wage rates (i.e., double and triple) and reduced labour availability (i.e., 50 person days per month), make them potentially useful in achieving the goals of the Environmental Land Management (ELM) Scheme in the United Kingdom and agri-environment schemes (AES) in the European Union and elsewhere.

## Supplementary Information

Below is the link to the electronic supplementary material.

Supplementary file1 : SFs Sensitivity Tests Figure (DOCX 63 KB)

Supplementary file2 : STEXTT Supplementary Text (DOCX 144 KB)

Supplementary file3 : SM1 Rectangular Field Algorithms (XLSX 88 KB)

Supplementary file4 : SM2 Non-Rectangular Field Algorithms (XLSX 461 KB)

## Data Availability

The data and all materials are available in the main manuscript or in the supplementary material sections. The LP coefficient excel spreadsheets subject to rectangular and non-rectangular 1 ha and 10 ha fields are available from the author by request at abdullahalamin@live.harper.ac.uk or abdullah.alamin@bau.edu.bd.
