# Supplementary material for: Economics of field size and shape for autonomous crop machines
Source: Precis Agric. 2023 Apr 9:1–28. Online ahead of print. doi: 10.1007/s11119-023-10016-w (PMC10103047; doi:10.1007/s11119-023-10016-w)
Supplement: Supplementary file 1 — Supplementary file1 : SFs Sensitivity Tests Figure (DOCX 63 KB) [file 11119_2023_10016_MOESM1_ESM.docx]

**SFig. 1:** Sensitivity test (i.e., wage rate double) for wheat unit cost of production in euro per ton for farms with rectangular fields of different sized farms. The labels on the data points for 1 ha and 10 ha fields are the size of the tractor used and the number of equipment sets. The curves without labels are the baseline analysis which was done without field size and shape modelling.

**SFig. 2:** Sensitivity test (i.e., wage rate triple) for wheat unit cost of production in euro per ton for farms with rectangular fields of different sized farms. The labels on the data points for 1 ha and 10 ha fields are the size of the tractor used and the number of equipment sets. The curves without labels are the baseline analysis which was done without field size and shape modelling.

**SFig. 3:** Sensitivity test (i.e., reduced labour availability of 50 person days per month) for wheat unit cost of production in euro per ton for farms with rectangular fields of different sized farms. The labels on the data points for 1 ha and 10 ha fields are the size of the tractor used and the number of equipment sets. The curves without labels are the baseline analysis which was done without field size and shape modelling.

**SFig. 4:** Sensitivity test (i.e., wage rate double) for wheat unit cost of production in euro per ton for farms with non-rectangular fields of different sized farms. The labels on the data points for 1 ha and 10 ha fields are the size of the tractor used and the number of equipment sets. The curves without labels are the baseline analysis which was done without field size and shape modelling.

**SFig. 5:** Sensitivity test (i.e., wage rate triple) for wheat unit cost of production in euro per ton for farms with non-rectangular fields of different sized farms. The labels on the data points for 1 ha and 10 ha fields are the size of the tractor used and the number of equipment sets. The curves without labels are the baseline analysis which was done without field size and shape modelling.

**SFig. 6:** Sensitivity test (i.e., reduced labour availability of 50 person days per month) for wheat unit cost of production in euro per ton for farms with non-rectangular fields of different sized farms. The labels on the data points for 1 ha and 10 ha fields are the size of the tractor used and the number of equipment sets. The curves without labels are the baseline analysis which was done without field size and shape modelling.
