# Supplementary material for: Economics of field size and shape for autonomous crop machines
Source: Precis Agric. 2023 Apr 9:1–28. Online ahead of print. doi: 10.1007/s11119-023-10016-w (PMC10103047; doi:10.1007/s11119-023-10016-w)
Supplement: Supplementary file 2 — Supplementary file2 : STEXTT Supplementary Text (DOCX 144 KB) [file 11119_2023_10016_MOESM2_ESM.docx]

Supplementary Materials for:

**Economics of field size and shape for autonomous crop machines**

**A. K. M. Abdullah Al-Amin^AB*^, James Lowenberg‑DeBoer^A^, Kit Franklin^A^, and Karl Behrendt^A^**

^A^Harper Adams University, Shropshire, Newport, TF10 8NB, UK

^B^Bangladesh Agricultural University, Mymensingh 2202, Bangladesh

*Correspondence to: [abdullahalamin@live.harper.ac.uk](mailto:abdullahalamin@live.harper.ac.uk) and [abdullah.alamin@bau.edu.bd](mailto:abdullah.alamin@bau.edu.bd)

**Supplementary Materials Encompass:**

1. Supplementary Text (i.e.., STEXTT Supplementary Text, which includes Main Text of the Technical Note).
2. Algorithms Spreadsheets (i.e., SM1 Rectangular Field Algorithms and SM2 Non-Rectangular Field Algorithms (i.e., Right-Angled Triangular Field))
3. Supplementary Figures (i.e., SFs Sensitivity Tests Figure, which includes Figures of the Sensitivity Tests).

**SUPPLEMENTARY TEXT:**

**A TECHNICAL NOTE ON AN ALGORITHM TO ESTIMATE FIELD TIMES AND FIELD EFFICIENCY**

Farm machinery performance evaluation has received substantial attention for the management of the arable crop farm and improvement of crop equipment operations (Bochtis et al., 2010; Sørensen and Nielsen, 2005). The development of Information and Communication Technology (ICT) systems accelerates the interest of maximizing operational efficiency (Grisso *et al.*, 2002; Grisso *et al.*, 2004; Bochtis *et al.*, 2010) and studies have suggested the shift from large conventional technology to autonomous machines for better performance, management and economic feasibility (Blackmore et al., 2005; Lowenberg-DeBoer et al., 2021; Shockley et al., 2019). To date, most of the research on autonomous machines concentrated on the technical feasibility of robotic systems (Duckett et al., 2018; Shamshiri et al., 2018). Very few production economics studies in arable robotic operations mentioned the significance of field performance, for instance, in automated (i.e., still required human operator) field operations, Clary *et al.* (2007) and Cembali *et al.* (2008) commented on the operational efficiency. Likewise, Sørensen *et al.* (2005) mentioned that efficiency is the critical prerequisite for improved profitability and assumed 80% efficiency for autonomous robotic weeding. Using the experience of the Hands Free Hectare (HFH) demonstration project at Harper Adams University, United Kingdom, Lowenberg-DeBoer *et al.* (2021) hypothesized that autonomous machines are more economical on small fields. They assumed constant 70% field efficiency for all operations and equipment sets based on the discussion of Witney (1988). However, the economics of autonomous crop machines over the range of field sizes and shapes has not been tested.

The existing conventional equipment and precision agriculture literature mainly developed models for predicting machinery performance (Sørensen, 2003; Sørensen and Nielsen, 2005; Jin and Tang, 2010; Fedrizzi *et al.*, 2019) and evaluated the field efficiency in farm operation (Taylor *et al.,* 2001; Grisso *et al.,* 2002; Taylor *et al.,* 2002; Grisso *et al.*, 2004; Bochtis *et al.*, 2010; Spekken and de Bruin, 2013). Nevertheless, the evaluation of field efficiency and field times of autonomous machines and the economic implications of field size and shape are still unexplored. To fill this research gap, the present study investigated the economics of autonomous machines in commodity crop production through the lens of machinery performance with a reference to different sized and shaped fields.

Considering the experiences of the HFH demonstration project, the study developed algorithms to estimate field times (h/ha) and field efficiency (%) for different sized rectangular and non-rectangular fields (i.e., right-angled triangular fields). The algorithms were adopted and modified following the study of Bochtis *et al.* (2010), Jin and Tang (2010), Shamshiri *et al.* (2013), Fedrizzi *et al.* (2019), Lowenberg-DeBoer *et al.* (2021) and the discussion of Witney (1988). The developed algorithms went beyond the existing studies as the present study used systems analysis from planting to harvesting that incorporated HFH on-field level demonstration experiences, field sizes and shapes, machine specifications, overlap consideration, headlands timing, interior passes timing, inputs refill timing, fuel refill timing, and field entry and exit time estimation.

The algorithms were developed considering the flexibility of future implications that would be applicable for any kinds of arable machines operated in different sized rectangular and non-rectangular fields. Keeping the research gaps in mind, the present study gave emphasis in the headlands and interior field time calculation as prior studies considered headlands passes and headlands turning as non-productive areas (Gónzalez *et al.,* 2007; Bochtis *et al.*, 2010). However, based on the experience of the HFH project, despite having lower yields compared to the rest of the field due to the compaction of soil and damage from machines turning, the headland is considered as a productive area. In addition, previous studies missed out pass to pass overlap (i.e., overlap percentage) in field efficiency estimation (Lowenberg-DeBoer et al., 2019). The study of Lowenberg-DeBoer (1999), Griffin et al., (2005), Ortiz *et al.* (2013) assumed 10% overlap as the benchmark.

Another contribution of the algorithms is that the present study addressed the limitations of earlier studies and incorporated their suggestions. Prior studies suggested that in field efficiency estimation, future studies should separately calculate the headlands turning time, and stoppages time (Taylor *et al.,* 2001; Taylor *et al.,* 2002; Bochtis *et al.*, 2010; Shamshiri *et al.*, 2013) because productive times and non-productive times play a significant role in field efficiency estimation (Bochtis et al., 2010; Jensen et al., 2015; Shamshiri et al., 2013; Spekken and de Bruin, 2013). Considering the significance of headlands turning types, field size and shape (for details see Jin and Tang, 2010 and Fedrizzi *et al.*, 2019), the study incorporated the headlands turning pattern of the HFH demonstration project. In summary, in developing the algorithms, the present study incorporated field and machine specifications, overlap percentage, productive times (i.e., field passes time, headlands turning time, and headlands passes time) and non-productive times (i.e., replenish inputs, refuelling, and blockages) for calculating the field efficiency of autonomous machines and conventional technologies with human operators for different sized and shaped fields.

Although logistics software is well developed in trucking and other transportation sectors (Software Advice, 2021), there is no readily available commercial software in the United Kingdom to estimate equipment times and field efficiency encompassing field and machine heterogeneity. Field times were sometimes generated as a by-product in the farm equipment path planning research literature (Hameed, 2014; Jensen et al., 2012; Oksanen and Visala, 2007; Spekken and de Bruin, 2013). The agri-tech economic studies often rely on text book (Lowenberg-DeBoer *et al.* 2021). For easy use of the algorithms by the students and researchers, especially those who are involved in the Agri-Tech Economics, the study developed algorithms in Excel spreadsheets. These algorithms could be further use for developing software and mobile app.

The technical note is organized with one section on the algorithms for the rectangular field and another section focused on the non-rectangular (i.e., right-angled triangular) field. The common assumptions and parameters used are as follows:

**Assumptions and Parameters Used**

The study assumed that the equipment enters the field from the entry side and completed the headlands first, after which the machine made usual flat turn to start the interior passes. The equipment ends on the entry side of the field, even if it is operating a partial swath or not at all on the return.

The field and HFH equipment specifications were collected from the experience of the HFH demonstration project at Harper Adams University, Newport, Shropshire, United Kingdom. HFH conventional machine with human operator and HFH autonomous machines are identical except for the autonomy hardware and software. The specifications of conventional machines were collected from Lowenberg-DeBoer *et al.* (2021), John Deere (<https://www.deere.co.uk/en/index.html>), Arslan et al. (2014), and Witney (1988). The parameters used and parameter definitions to calibrate the algorithms are presented in Table S1 and Table S2.

**ALGORITHMS TO ESTIMATE FIELD TIMES AND FIELD EFFICIENCY FOR RECTANGULAR FIELD**

**Estimation of Field Times and Field Efficiency**

The algorithms used for estimating field times (h/ha) and field efficiency (%) for different sized rectangular fields are sub divided in the following sections:

1. The first section incorporated the main parameters and base calculations, which encompassed field and equipment specifications (for details see Table S1 and algorithms presented in excel spreadsheet in Supplementary Material i.e., SM1 Rectangular Field Algorithms).
2. Secondly, the algorithms incorporated the headland area and field time calculation.
3. Thirdly, interior field and passes time were estimated.
4. Fourthly, non-productive time calculations were performed that incorporated headland and interior field input refill time, fuel refill time, and blockages times. By summing the input and fuel refilling time and blockages the total non-productive time (i.e., total stoppages times) were calculated.
5. Fifthly, the algorithms estimated the total field operation time.
6. Sixthly, the theoretical field time was calculated based on the machine design specifications, and
7. Finally, field efficiency was estimated as the ratio of theoretical field time based on machine design specifications like the estimates of theoretical field time to its actual field productivity.
8. **Main Parameters and Base Calculations**

***Field attributes calculation***

The study tested the algorithms for 1ha, 10ha, 20ha, 50ha, 75ha, and 100ha rectangular fields (where, 1 ha = 10,000 m^2^) equipped with 28 kW conventional machines with human operator and autonomous machines, and 112 kW and 221 kW conventional machines with human operators. Details of the field specifications are given in Table S1 and Supplementary Material (i.e., SM1 Rectangular Field Algorithms). The study assumed that length of the field is ten times the width of the field. Following this assumption, the width of the rectangular field was estimated as the square root of the area divided by ten:

$W_{f}=Sqrt (A/10)$ … … (1)

where, $W_{f}$ is the width of the field, and A is the area of the field.

Subsequently, the length of the rectangular field was calculated as:

$L_{f}=10*W_{f}$ … … (2)

where, $L_{f}$ is the length of the field.

The equipment specifications are evident in the Table S1 (for further details see the algorithms in the excel spreadsheet in Supplementary Material i.e., SM1 Rectangular Field Algorithms). The effective swath width of the implement was calculated as the width of the implement multiplied by one hundred minus overlap percentage:

$W_{s}=W_{m}*((100-Overlap percentage)/100)$ … … (3)

where, $W_{s}$ is the effective swath width, and $W_{m}$ is the width of the implement. For all operations and equipment sets this study assumed a 10% overlap percentage following Lowenberg-DeBoer et al. (2021).

The total number of passes around the headlands for a rectangular field was calculated as the width of the headland divided by the effective swath width of the implement:

$N_{hp}= \left\lceil W_{h}/W_{S} \right\rceil$ … … (4)

where, $N_{hp}$ is the total number of passes around the headland to the nearest positive integer.

This algorithm ensures that the headland width allows operation of all equipment sets, and $W_{h}$ is the width of the headland (i.e., the study assumed headland width equals the effective swath width of the sprayer as the sprayer width was the largest implement among drilling, spraying, and harvesting operations).

1. **Headlands Area and Field Time Calculation**

Time for the headland round was calculated as the sum of the length and width of the field, minus the width of the previous headland rounds in four sides of the field, minus the square corner distance of four corners of the rectangular field, which is divided by the implement running speed in the passes, and plus the four corners distances that is divided by the turning speed of the implement. The turning speed of the implement was one third of the implement running speed in the passes. Thousands were used to convert speed from km/h to m/h. The study used the following algorithm to calculate headlands round time:

$$T_{\mathrm{hi}}=\left\{ \begin{aligned} (((L_{f}+Wf) -{(n}_{hi}-1)*4*W_{S})-((2*r)+(0.5*2*W_{s}))*2)*2/(1000*v_{p}) \\ + (r*\pi/2)*4/(1000*v_{t}) ;n_{hi}\leq N_{hp} \\ 0; n_{hi}>N_{hp} \end{aligned} \right.$$

… … (5)

where, $T_{hi}$ is the time required for the headland round (i = 1, 2, … …, 9), $n_{hi}$is the number of rounds required for the headland operation (i. e., $n_{hi}$ =1, 2, … …, 9), *r* is the turning radius of the implement, $v_{p}$ is the implement running speed in the passes, and $v_{t}$ is the implement turning speed. When the number of passes is less than or equal to the total number of headlands passes, the first term is half the perimeter of the field. It is multiplied by 2 (the 2 just before the first slash) to give the whole perimeter. In the first headland round the machine travels a path that is half a swath width inside the perimeter; this is why $(0.5*2*W_{s})$ is deducted. For each headland round after the first another four swath widths are deducted in each half perimeter (i.e., ${(n}_{hi}-1)*4*W_{S})$; one on each side of the field width and one on each side of the field length. The corner turn distance is as the turning speed and in estimated in the second term which is four quarter turns in a half field perimeter $(i.e., (r*\pi/2)*4)$. To avoid double counting two radius lengths are deducted from the first term at pass speed (i.e., 2*r). To allow for deceleration and acceleration before and after the corner a second swath width is deducted $(0.5*2*W_{s})$.

After completing the headlands first, the machine entered the interior field (i.e., refers to the field excluding width of the headlands on all four-sides). The time from headland to the first interior pass was calculated following the experience of the HFH demonstration project and the study adopted and modified the typical “Flat” turn of Jin and Tang (2010) as follows:

$T_{\mathrm{hturn}}=(W_{s}*(1 + n + Cot\delta)) + (r*(\pi-2)) / (1000*v_{t})$… … (6)

where$,T_{hturn}$ is the turning time from headland to first interior pass, *n* is the number of swaths skipped during turning, and $\delta$ is the swath direction in radians. The Jin and Tang (2010) equation is modified by including the “n” which is the linear distance along the headland when swaths are skipped before resuming the next pass. Skipping swaths is required for flat turns when the turning radius exceeds the swath width. The $W_{s}$*Cot$\delta$ term is used to estimate the extra distance travelled when the headlands are not exactly perpendicular to the passes.

The total time in the headlands rounds was estimated as the summation of all headlands round times and the turning time from headland to first interior pass as follows:

$T_{h}= = \sum_{i}^{9} T_{hi} + T_{hturn}$ … … (7)

where, $T_{h}$ is the total time in the headland rounds.

The area of the headland of the field was calculated as follows:

$A_{hf}=A-(L_{if}*W_{if})$ … … (8)

where, $A_{hf} i$s the area of the headland, $L_{if}$is the length of the interior field, and $W_{if}$ is the width of the interior field.

1. **Interior Field and Passes Time Calculation**

Interior field refers to the field excluding the four-sided widths of the headland. The length of the interior field was calculated as the length of the field minus the two sides headlands width of the field:

$L_{if}=\left\{ \begin{aligned} {(L}_{f} -{2*W}_{h});W_{if}\geq W_{s} \\ {0; W}_{if}<W_{s} \end{aligned} \right.$ … … (9)

The width of the interior field was calculated as the width of the field minus the two sides headlands width of the field as follows:

$W_{if} = \left\{ \begin{aligned} \left( W_{f}-{2*W}_{h} \right); (W_{f}-{2*W}_{h})>0 \\ 0;\left( W_{f}-{2*W}_{h} \right)\leq0 \end{aligned} \right.$ … … (10)

The area of the interior field was estimated as the length of the interior field multiplied by the width of the interior field:

$A_{if}=L_{if}*W_{if}$ … … (11)

where, $A_{if}$ is the area of the interior field.

For rectangular field, the number of interior headland turn was estimated by dividing the width of the interior field parallel to which the interior turns take place by the effective swath width as follows:

$N=\left\{ \begin{aligned} \left\lceil W_{if}/W_{s} \right\rceil; \left\lceil W_{if}/W_{s} \right\rceil>0 \\ 0; \left\lceil W_{if}/W_{s} \right\rceil\leq0 \end{aligned} \right.$ … … (12)

where, N is the total number of interior headlands turn. This algorithm ensures that the headland width allows operation of all equipment sets.

The total number of interior passes of the field must be even to bring the machine back to the entry side of the field. Consequently, it was estimated as the total number of interior headlands if even and total number plus one if odd.

$N_{P} =\left\{ \begin{aligned} N+1;N=ODD \\ N;N=EVEN \end{aligned} \right.$ … … (13)

where, $N_{P}$ is the total number of interior passes. The conditional algorithm is used to ensure field entry and exit in the same path.

The total time in the interior field passes was calculated by multiplying the length of an interior field pass with the total number of interior field passes which is divided by the running speed of the implement in the passes and one thousand is divided to reach in the unitary of the units used:

$T_{p}= (N_{P}*L_{if}) / (1000*v_{p})$… … (14)

where, $T_{p}$ is the total time in the interior field passes.

In field efficiency calculation, the headland turning time is considered with greater importance (Witney, 1988; Grisso *et al.*, 2002; Gónzalez *et al.,* 2007; Bochtis *et al.*, 2010; Jin and Tang, 2010). Even though, prior studies considered headlands as non-productive area (Witney, 1988; Gonzalez *et al.,* 2004; Gónzalez *et al.,* 2007; Bochtis *et al.*, 2010), the study treated headland as useful area based on the HFH demonstration experience. The methodology of headland turning time was adopted and modified from the study of Jin and Tang (2010) and Fedrizzi *et al.* (2019). The study of Jin and Tang (2010) considered several turning types (i.e., “Flat” turn, “U” turn, “Bulb” turn, “Hook” turn). The study modified their algorithm following the HFH demonstration project, which always follow the “Flat” turn, unlike the “Flat” turn of Jin and Tang (2010) as HFH equipment skipped swaths. The autonomous machinery operations of HFH followed the “Flat” turn with skipping of swaths (i.e., during headlands turning the machine skipped two swaths nearer and enter the field after skipping those swaths) (for typical “flat turn” see Jin and Tang 2010 and for HFH “flat turn” see Fig.1 in the main manuscript). With the experience of the HFH demonstration project, the turning time for “flat” turn goes beyond the calculation of Jin and Tang (2010) and was calculated as:

$T_{\mathrm{turn}}= ((W_{s}*(1 + n + Cot\delta)) + (r*(\pi-2)) / (1000*v_{h})$ … … (15)

where, $T_{turn}$ is the interior headland turning time.

The total interior headlands turning time of the field was estimated by multiplying the number of interior headlands turn with the interior headlands turning time as follows:

$T_{r}=N *T_{turn}$ … … (16)

where, $T_{r}$ is the total interior headlands turning time.

The distance to field entry and exit assumes that after the last pass (and returning to the entry side of the field if the number of passes in odd) the machine ends up at the far side of the interior field relative to the entry. The distance from the far side is assumed to be a diagonal line across the headland to the entry in the corner. Using the Pythagorean theorem that distance is the square root of the square of the headland width plus the square of the field width minus the headland width. To calculate the total time for field entry and exit passes these square distances of field entry and exit were divided by the implement turning speed. The algorithm of the total time for field entry and exit passes was as follows:

$T_{fe}= \surd(({W_{h})}^{2}+ ({W_{f}-W_{h})}^{2}) / {(1000*v}_{t})$ … … (17)

where, $T_{fe}$ is the total time for field entry and exit passes.

The total observed time in the interior field and passes ($T_{obs}$) incorporated the total time in the interior field passes ($T_{p}$), the total time in the interior headlands turning ($T_{r}$), and total time for field entry and exit passes ($T_{fe}$) as follows:

$T_{obs}= T_{p} + T_{r} + T_{fe}$ … … (18)

1. **Non-Productive Times Calculation**

The non-productive time is another important factor associated with field times and field efficiency estimation. In this study, non-productive time encompassed replenishing inputs, blockage, and refueling. The algorithms for estimating non-productive times are as follows:

***Headland and interior field input refill time calculation***

Input required for the interior field was calculated as multiplication of the seeding rate per ha to the area of the interior field as follows:

${IR_{if}=(Q}_{s}*A_{if})/10000$ … … (19)

where, ${IR}_{if}$ is the input required for the interior field, and $Q_{s}$ is the seeding rate per ha.

Input required for the headlands was calculated as multiplication of the seeding rate per ha to the area of the headland of the field:

${IR_{h}=(Q}_{s}*A_{hf})/10000$ … … (20)

where, ${IR}_{h}$ is the input required for the headland.

Input required for the headland and interior fields was calculated by summing the input required for the interior field and input required for the headland as follows:

${IR}_{hif}=IR_{if}+ IR_{hf}$ … … (21)

where, ${IR}_{hif}$ is the input required for the headland and interior field.

Number of refills needed within the field was estimated as dividing the input required for the headland and interior field to the capacity of the bin minus one. Because the study assumed that the equipment entered the field and started operation with a full bin of seed, consequently, the number of refills needed “within” the field would be less than the total number of refills needed to complete the field operation.

$NRN_{f}= \left\lceil\left\lfloor(({IR}_{hif}/C_{b})-1) \right\rfloor\right\rceil$ … … (22)

where, $NRN_{f}$ is the number of refills needed “within” the field, $C_{b}$ is the capacity of the bin. This algorithm used $ROUNDDOWN or ROUNDUP$represented with $\left\lceil\left\lfloor\right\rfloor\right\rceil,$which allows all operations with all equipment sets. ROUNDDOWN is used based on the consideration of the capacity of the bin (i.e., if the sum of the input requirement is less than the bin capacity). However, if the sum of the input requirement is greater than the capacity of the bin, the study used ROUNDUP to avoid equipment running with empty bin and ensure seed in the bin.

Total stoppage time for input refill was estimated by multiplying the number of refills needed “within” the field with the stoppage time for a single input refill which is divided by 60 considering unitary of the units used in hour:

$T_{sir}= {(T}_{ssir}/60)* NRN_{f}$ … … (23)

where, $T_{sir} i$s the total stoppage time for input refill, $T_{ssir}$is the stoppage time for a single input refill.

***Headland and interior field fuel refill time calculation***

The headland and interior field fuel refill time followed the same estimation procedures mentioned in the above equation (19), (20), (21), (22), and (23). In this case, for estimating the fuel required for the interior field and headland, the fuel consumption rate per ha ($Q_{f}$) was used. Likewise, to calculate the number of refills needed “within” the field the capacity of the fuel tank ($C_{t})$ was considered. Finally, using the same algorithms of stoppage time calculation the total stoppages time for fuel refill ($T_{sfr}$) was calculated, where the stoppage time for single fuel refill ${(T}_{ssfr})$ was considered.

***Blockages time calculation***

They study assumed zero blockage time because of lack of clear data from the HFH demonstration project.

***Total stoppages time calculation***

The total stoppages time “within” the field is the summation of the total stoppages time for input refill ($T_{sir}$), total stoppage time for blockage ($T_{sb}$), and total stoppages time for fuel refill ($T_{sfr}$) that was calculated as:

$T_{sf} = T_{sir}+ T_{sb}+ T_{sfr}$ … … (24)

where, $T_{sf}$ is the total stoppages time “within” the field.

1. **Total Field Operation Time Calculation**

The total time for field operation was calculated as the summation of the total observed time in the interior field and passes ($T_{obs}$), total headland round time ($T_{h}$), and the total stoppages time in the field ($T_{sf}$)$:$

$T_{tfo}= T_{obs}+T_{h}+T_{sf}$ … … (25)

where, $T_{tfo}$ is the total time for field operation.

Total time for field operation per hectare was estimated as the ratio of the total time for field operation by the area of the field in hectare.

$T_{tfoha}=T_{tfo}/A$ … … (26)

where, $T_{tfoha}$ is the total time for field operation per hectare.

1. **Theoretical Field Time Calculation**

The theoretical field time was measured based on the machine design specifications. The study followed Lowenberg-DeBoer et al. (2021) to estimate theoretical field time as follows:

$T_{T}=[A / (W_{Ts}*v_{p}*1000)]$… … (27)

where, $T_{T}$ is the theoretical field time, and $W_{Ts}$ is the theoretical swath width.

Theoretical field time per hectare was estimated as the ratio of the theoretical field time divided by the field area in hectare.

$T_{Tha}=T_{T}/A$ … … (28)

where, $T_{Tha}$ is the theoretical field time per hectare.

1. **Field Efficiency Calculation**

The study calculated field efficiency, following the estimation procedure of Lowenberg-DeBoer *et al.* (2021), Bochtis *et al.* (2010), and Shamshiri *et al.* (2013). In the present study, field efficiency is defined as the ratio of theoretical field time based on machine design specifications like the estimates of theoretical field time to its actual field productivity:

$E_{f}= [T_{T} / (T_{obs}+ T_{h} + T_{\mathrm{sf}})] * 100$… … (29)

where, $E_{f}$ is the field efficiency, T_T_ is the theoretical field time, $T_{\mathrm{obs}}$ is the total observed time in the interior field and passes, $T_{h}$ is the total headland round time, and $T_{sf}$ total stoppages time “within” in the field. Simply the above field efficiency algorithm can be represented as: $T_{tfoha}/T_{Tha}$ (see the algorithms in the excel spreadsheet in Supplementary Material i.e., SM1 Rectangular Field Algorithms).

**ALGORITHMS TO ESTIMATE FIELD TIMES AND FIELD EFFICIENCY FOR RIGHT-ANGLED TRIANGULAR FIELD**

**Estimation of Field Times and Field Efficiency**

The algorithms used for estimating the field times (h/ha) and field efficiency (%) for different sized non-rectangular (i.e., right-angled triangular) fields are also sub divided in seven sub-sections similar to the rectangular field algorithms. Details of the algorithms are as follows:

1. **Main Parameters and Base Calculations**

***Field attributes calculation***

To test the algorithms, the study used 1 ha, 10 ha, 20 ha, and 25 ha sized right-angled triangular fields equipped with the same equipment sets used in rectangular fields. Details of the field specifications are given in Table S1 and Supplementary Material (i.e., SM2 Non-Rectangular Field Algorithms). The study assumed that each field has the height equalling twice the base. Following this assumption, the adjacent base of the right-angled triangular field was calculated as the square root of the area:

${Ab}_{f}=Sqrt A$… … (1)

where, ${Ab}_{f}$ is the adjacent base of the field.

As the study assumed that height equalling twice the base, the opposite height of the triangular field was calculated as:

${Oh}_{f}= 2*{Ab}_{f}$… … (2)

where, ${Oh}_{f}$ is the opposite height of the field.

Following the Pythagorean theorem, or Pythagoras' theorem, the hypotenuse of the right-angled triangular field was calculated as:

${Hyp}_{f}=Sqrt (({{Oh}_{f})}^{2}+({{Ab}_{f})}^{2})$… … (3)

where, ${Hyp}_{f}$ is the hypotenuse of the field.

Right angle of the triangle was calculated as:

$\theta= Degrees ((PI()/2) = Radians (Degrees)$… … (4)

where, $\theta$ is the right angle of the right-angled triangular field. During the calculation of angles, to ensure naturalness in mathematics, trigonometric arcs relationship, and have more elegant formulation of a number, the degrees (^o^) were converted to radian (rad) considering the International Systems of Units.

Second largest angle of the right-angled triangular field was estimated using the sine trigonometric function of an angle, where sine is the ratio of the opposite height to the hypotenuse:

$\varphi= Degrees (Asin ({Oh}_{f}/{Hyp}_{f})) = Radians (Degrees)$… … (5)

where, $\varphi$ is the second largest angle$.$

Smallest angle of the right-angled triangular field was estimated using the sine trigonometric function of an angle:

$\mu= Degrees (Asin ({Ab}_{f}/{Hyp}_{f}) = Radians (Degrees)$… … (6)

where, $\mu$ is the smallest angle$.$

In case of right-angled triangular fields, the study assumed same algorithms similar to rectangular field to calculate effective swath width ($W_{s})$ and the total number of passes around the headland${(N}_{hp}).$For details of the equipment specifications and estimation see Table S1 and Supplementary Material (i.e., SM2 Non-Rectangular Field Algorithms).

1. **Headlands Area and Field Time Calculation**

Time for the headland round was calculated as the sum of the opposite height, adjacent base, and hypotenuse of the right-angled triangular field, minus the width of the previous headland rounds in three sides, minus the square corner distance of the three corners of the right-angled triangular field, which is divided by the implement running speed in the passes, and plus the three corners distances that is divided by the implement turning speed. When the number of passes is less than or equal to the total number of headland passes, the first term is the perimeter of the field. The corner estimates are multiplied by 3 which is before the first slash to give the whole perimeter. In the first headland round the machine travels a path that is half a swath width inside the perimeter; this is why $(0.5*2*W_{s})$ is deducted. For each headland round after the first another three swath widths are deducted in each half perimeter (i.e., ${(n}_{hi}-1)*3*W_{S})$; on the opposite height, adjacent base, and hypotenuse of the right-angled triangular field. To avoid double counting two radius lengths are deducted from the first term at pass speed (i.e., 2*r). The study used the following algorithm to calculate headlands round time:

$$T_{hi}=\left\{ \begin{aligned} (({{Oh}_{f}+Ab}_{f}+{Hyp}_{f})-{(n}_{hi}-1)*3*W_{S})-((2*r) + (0.5*2*W_{s})))*3/(1000*v_{p}) \\ +(\left( r*\theta\right)+\left( r*\varphi\right)+\left( r*\mu\right))/(1000*v_{t});n_{hi} \leq N_{hp} \\ 0;n_{hi} \leq N_{hp} \end{aligned} \right.$$

… … (7)

After completing the headlands first, the machine entered in the interior field. The time from headland to the first interior pass ($T_{\mathrm{hturn}}$) and the total time in the headland rounds $(T_{h})$was calculated following the same algorithm described in rectangular field algorithm section.

The area of the headland was calculated as follows:

$A_{hf}=A-(0.5*A_{bif}*Oh_{if})$ … … (8)

where, $A_{hf}$ is the area of the headland, ${Ab}_{if}$ is the adjacent base of the interior field, and ${Oh}_{if}$ is the opposite height of the interior field.

**Interior Field and Passes Time Calculation**

For right-angled triangular field, interior field refers to the field excluding three-sided width of the headlands. The adjacent base of the interior field indicated the whole adjacent base of the external field minus the headland width on the right-angled corner side minus the horizontal widths across the diagonal headland that was represented by sine function of an angle and minus the horizontal width in the headland corner which was represented by the tangent function of an angle. The sine is the ratio of the opposite height to the hypotenuse and the tangent is the ratio of the opposite height to the adjacent base. The adjacent base of the interior triangular field was estimated as follows:

${Ab}_{if}= {Ab}_{f} - W_{h} - (W_{h}/Sin(\varphi)) - (W_{h}/Tan(\varphi))$… … (9)

where, ${Ab}_{f}$ is the adjacent base of the field, $W_{h}$ is the width of the headland, and $\varphi$ is the second largest angle.

Projection of the width of the headland on the hypotenuse that is close to the smallest angle of the right-angled triangular field was calculated as the ratio of the width of the headland to the cos trigonometric function of an angle, where cos is the ratio of the adjacent base to the hypotenuse:

$P_{Whhyp}= W_{h}/Cos\left( \varphi\right)$… … (10)

where, $P_{Whhyp}$ is the projection of the width of the headland on the hypotenuse.

Opposite height of the interior triangular field is the opposite height of the field minus the projected width of the headland on the hypotenuse minus width of the headland, minus the ratio of the width of the headland to the tan trigonometric function of the smallest angle was estimated as:

${Oh}_{if} = {Oh}_{f} - P_{Whhyp} - W_{h} - ((W_{h}/\mathrm{Tan}(\mu))$… … (11)

The number of interior headlands turn that were taken parallel to the adjacent base and the hypotenuse of the external field was estimated as follows:

$N=\left\lceil({Ab}_{if}/W_{s}-1, 0) \right\rceil$ … … (12)

where, N is the total number of interior headlands turn, $W_{s}$ is the effective swath width. This algorithm ensures that the headland width allows operation of all equipment sets.

The total number of interior passes ($N_{p}$) was calculated following the same process described in the rectangular field algorithm section (for further details see the Supplementary Material i.e., SM2 Non-Rectangular Field Algorithms).

To calculate the length of the interior field passes, the study subtracted half of the effective swath width from the adjacent base of the interior triangular field minus the width of the previous headland rounds and multiply this with the tangent function of the second largest angle. The length of the interior field passes was calculated as:

$l_{pi}=\left\{ \begin{aligned} ABS(({Ab}_{if}-W_{S}/2) -({(n}_{hi}-1)*W_{S})*Tan (\varphi), 0); n_{pi}\leq N_{p} \\ 0; n_{pi}>N_{p} \end{aligned} \right.$ … … (13)

where, $l_{pi}$ is the length of the interior field passes (i = 1, 2, … …, 361), $n_{pi}$is number of passes in the interior field operation (i. e., $n_{pi}$ =1, 2, … …, 361), $N_{p}$ is the total number of interior field passes, and ABS is used for absolute value function as the study assumed that the equipment ends on the entry side of the field, even if the equipment is operating in the small end passes (i.e., to deal with the negative distance in case of tiny end passes).

The total length of the interior field passes was estimated as the summation of the length of all interior field passes as follows:

$L_{p}$= $\sum l_{pi}$ … … (14)

where, $L_{p}$ is the total length of the interior passes.

The total time in the interior field passes was calculated by dividing the total length of the interior field passes to the running speed of the implement in the passes as follows:

$T_{p}$= $\sum L_{P}$/(1000*$V_{p}$) … … (15)

For interior headlands turning time ($T_{turn}$) and total interior headlands turning time ($T_{r}$) calculation, the study used the same algorithms described in the rectangular field algorithms section.

Width of the headland with the second largest angle was calculated as follows, where the adjacent base of the interior triangular field and the headland width on the right-angled corner side was subtracted from the adjacent base of the exterior field:

$W_{h2ndlc}$ = ${Ab}_{f}$ - ${Ab}_{if}$ - $W_{h}$ … … (16)

where, $W_{h2ndlc}$ is the width of the headland on the second largest corner.

The distance for field entry and exit encompassed the square of the travel distance of the headland width on the right-angled corner side and the headland width on the second largest corner side plus these square corner headlands widths were subtracted from the square of the adjacent base of the external field. To calculate the total time for field entry and exit, these square distances of field entry and exit were divided by the implement turning speed. The algorithm of the total time for field entry and exit passes was as follows:

$T_{fe}= Sqrt ((W_{h} + {W_{h2ndlc})}^{2} + ({Ab}_{f} - (W_{h} + {W_{h2ndla}))}^{2}/{(1000*v}_{t})$ … … (17)

The total observed time in the field and passes ($T_{obs}$) was calculated using the same procedure described in the rectangular algorithm section.

1. **Non-Productive Times Calculation**

The non-productive time encompassed replenishing inputs, blockage, and refueling.

Input required for the interior field ($IR_{if}$) was calculated as multiplication of the seeding rate per ha ($Q_{s}$) with the total length of the interior passes ($L_{p}$) and effective swath width ($W_{s}$) as follows:

${IR_{if}=(Q}_{s}*L_{p}*W_{s})/10000$ … … (18)

The rest of the calculation such as input required for the headlands $(IR_{h})$, input required for the headland and interior fields (${IR}_{hif}$), number of refills needed within the field ($NRN_{f}),$ and time stoppage for input refill ($T_{sir}$) followed the same estimation procedures mentioned in the rectangular algorithms’ sections.

For the calculation of headland and interior field fuel refill time and total stoppages time ($T_{sf})$the study used the same procedure described above and in the rectangular algorithms section.

For estimating other sub-sections (5, 6, and 7) which encompass the calculation of total time for field operation ($T_{tfo}$), theoretical field time ($T_{T}$), and estimation of field efficiency ($E_{f}),$ the study followed the same estimation procedures described earlier in rectangular algorithms sections (for details see excel spreadsheet in the Supplementary Material i.e., SM2 Non-Rectangular Field Algorithms).

**Validation of Field Efficiency Estimation**

Even though field efficiencies are not constant values that may vary for specific equipment and depends on various factors (Bochtis et al., 2010; Hunt, 2001), to validate the algorithms, the study provides the following field efficiency comparison as shown in Table S3. The field efficiency estimation is justifiable based on the comparison of Witney (1988) and Hunt (2001). The estimates available in the literature are few decades earlier and unable to address field and equipment heterogeneity, whereas the present study provides the recent experience of field efficiency considering field and equipment heterogeneity. Future attempts should be made to validate with on-field estimation of autonomous machines and conventional machines with human operators. Because the Hands Free Hectare (HFH) was a demonstration project, it was difficult to separate on-field stops and down time while the engineers tinkered from those stoppage that would have occurred in normal field operations. Consequently, the model parameters were based on published machine specifications and farm budget information, and guided the experience of the HFH project demonstrated at Harper Adams University, Newport, Shropshire, United Kingdom.

**Conclusions**

Field efficiency maximization is an important consideration in arable field operations. The study developed algorithms for estimating field times (h/ha) and field efficiency (%) of different sized and shaped rectangular and non-rectangular (i.e., right-angled triangular) fields equipped with autonomous machines and conventional machines with human operators. The ultimate objective of the study was to examine the economics of autonomous machines subject to field size and shape with the lens of field efficiency and field times. The study is the first attempt in the development of algorithms for autonomous and conventional machine for arable field operations from planting to harvesting. The calculated field efficiencies were used to estimate the equipment times that were used as an input for estimating the coefficient of labour, tractor, and combine used for Hands Free Hectare - Linear Programming (HFH-LP) model. The coefficient estimation format is available in the supplementary material of Lowenberg-DeBoer et al. (2021), namely field operations and equipment times by crop, month and equipment set of optimum yields. The assessment of the economic implications will guide the farmers, engineers, agribusinesses, and policy makers for further development of the technology, decision making for farm management and machinery selection with the existing farm resource constraints.

References of the Algorithms of Field Times and Field Efficiency

Arslan, S., Anna, M.P., Emily, S., Zisis, T., Vitor, G., David, W., & Richard, G. J. (2014). *Fuel consumptions and draft power requirements of three soil tillage methods and three field traffic systems.* Paper 1900051, ASABE, St Joseph, Michigan, USA.

Blackmore, S., Stout, B., Wang, M., & Runov, B. (2005). *Robotic agriculture - the future of agricultural mechanisation?* Conference: European Conference on Precision Agriculture, pp. 621-628.

Bochtis, D. D., Sørensen, C. G., Green, O., Moshou, D., & Olesen, J. (2010). Effect of controlled traffic on field efficiency. *Biosystems Engineering*, *106*(1), 14–25. https://doi.org/10.1016/j.biosystemseng.2009.10.009

Cembali, T., Folwell, R. J., Clary, C. D., & Mari, M. (2008). Economic comparison of selective and nonselective mechanical harvesting of asparagus. *International Journal of Vegetable Science*, *14*(1), 4–22. https://doi.org/10.1080/19315260801890476

Clary, C. D., Ball, T., Ward, E., Fuchs, S., Durfey, J. E., Cavalieri, R. P., & Folwell, R. J. (2007). Performance and economic analysis of a selective asparagus harvester. *Applied Engineering in Agriculture, 23(5)*, 571–577. https ://doi.org/10.13031/2013.23665.

Duckett, T., Pearson, S., Blackmore, S., & Grieve, B. (2018). Agricultural Robotics: The Future of Robotic Agriculture.UK-RAS White Papers, EPSRC UK-Robotics and Autonomous Systems Network. Retrieved September 2, 2018, from https://arxiv.org/ftp/arxiv/papers/1806/1806.06762.pdf

Fedrizzi, M., Antonucci, F., Sperandio, G., Figorilli, S., Pallottino, F., & Costa, C. (2019). An artificial neural network model to predict the effective work time of different agricultural field shapes. *Spanish Journal of Agricultural Research*, *17*(1), 1–9. https://doi.org/10.5424/sjar/2019171-13366

Gonzalez, X. P., Alvarez, C. J., & Crecente, R. (2004). Evaluation of land distributions with joint regard to plot size and shape. *Agricultural Systems*, *82*(1), 31–43. https://doi.org/10.1016/j.agsy.2003.10.009

Gónzalez, X. P., Marey, M. F., & Álvarez, C. J. (2007). Evaluation of productive rural land patterns with joint regard to the size, shape and dispersion of plots. *Agricultural Systems*, *92*(1–3), 52–62. https://doi.org/10.1016/j.agsy.2006.02.008

Griffin, T., Lambert, D., & Lowenberg-DeBoer, J. (2005). Economics of lightbar and auto-guidance GPS navigation technologies. *Precision Agriculture, ECPA 2005*, (January 2005), 581–587.

Grisso, R. D., Jasa, P. J., & Rolofson, D. E. (2002). Analysis of traffic patterns and yield monitor data for field efficiency determination. *Journal of Agricultural Safety and Health*, *18*(2), 171–178. https://doi.org/10.13031/2013.7782

Grisso R. D., Kocher M. F., Adamchuk V. I., Jasa P. J., & Schroeder M. A. (2004). Field Efficiency Determination Using Traffic Pattern Indices. *Applied Engineering in Agriculture*, *20*(5), 563–572. https://doi.org/10.13031/2013.17456

Grisso R.B., Jasa, P.J., Schroeder, M.A., Kocher, M.F., & Adamchuk. V.I. (2002). *Field Efficiency Influences From Steering Adjustments Using Analysis of Traffic Patterns.* ASAE Meeting Paper No. 021009. ASAE, 2950 Niles Road, St. Joseph, MI 49085-9659 USA., (August 2002). https://doi.org/10.13031/2013.9717

Hameed, I. A. (2014). Intelligent coverage path planning for agricultural robots and autonomous machines on three-dimensional terrain. *Journal of Intelligent and Robotic Systems: Theory and Applications*, *74*(3–4), 965–983. https://doi.org/10.1007/s10846-013-9834-6

High Level Panel of Experts (HLPE). (2013). *Investing in smallholder agriculture for food security.* FAO. Food and Agriculture Organization of the United Nations, (6), 112.

Hunt, D. (2001). *Farm Power and Machinery Management.* Iowa State University Press, Ames, Iowa, USA.

Jensen, M. A. F., Bochtis, D., Sorensen, C. G., Blas, M. R., & Lykkegaard, K. L. (2012). In-field and inter-field path planning for agricultural transport units. *Computers and Industrial Engineering*, *63*(4), 1054–1061. https://doi.org/10.1016/j.cie.2012.07.004

Jensen, M. F., Nørremark, M., Busato, P., Sørensen, C. G., & Bochtis, D. (2015). Coverage planning for capacitated field operations, Part I: Task decomposition. *Biosystems Engineering*, *139*, 136–148. https://doi.org/10.1016/j.biosystemseng.2015.07.003

Jin, J., & Tang, L. (2010). Optimal coverage path planning for arable farming on 2D surfaces. *Transactions of the ASABE*, *53*(1), 283–295.

Lowder, S. K., Skoet, J., & Raney, T. (2016). The Number, Size, and Distribution of Farms, Smallholder Farms, and Family Farms Worldwide. *World Development*, *87*, 16–29. https://doi.org/10.1016/j.worlddev.2015.10.041

Lowenberg-DeBoer, J. (1999). GPS based guidance systems for agriculture. In *Purdue Agricultural Economics Report.* (p. Purdue University, December, 8-9. https://ag.purdu).

Lowenberg-DeBoer, J., Behrendt, K., Godwin, R., & Franklin, K. (2019). The Impact of Swarm Robotics on Arable Farm Size and Structure in the UK. *Agricultural Economics Society Annual Conference*, (April). https://hau.collections.crest.ac.uk/17421/

Lowenberg-DeBoer, J., Franklin, K., Behrendt, K., & Godwin, R. (2021). Economics of autonomous equipment for arable farms. Precision Agriculture, 22, 1992–2006. https://doi.org/10.1007/s11119-021-09822-x

Oksanen, T., & Visala, A. (2007). Path Planning Algorithms for Agricultural Machines. *Agricultural Engineering International the CIGR Ejournal*, *IX*(31), 1–19.

Ortiz, B. V., Balkcom, K. B., Duzy, L., van Santen, E., & Hartzog, D. L. (2013). Evaluation of agronomic and economic benefits of using RTK-GPS-based auto-steer guidance systems for peanut digging operations. *Precision Agriculture*, *14*(4), 357–375. https://doi.org/10.1007/s11119-012-9297-y

Shamshiri, R., Ehsani, R., Maja, J. M., & Roka, F. M. (2013). Determining machine efficiency parameters for a citrus canopy shaker using yield monitor data. *Applied Engineering in Agriculture*, *29*(1), 33–41. https://doi.org/10.13031/2013.42526

Shamshiri, R. R., Weltzien, C., Hameed, I.A., Yule, I.J., Grift, T.E., Balasundram, S. K., Pitonakova, L., Ahmad, D., & Chowdhary, G. (2018). Research and development in agricultural robotics: A perspective of digital farming. *International Journal of Agricultural and Biological Engineering*, *11*(4), 1–11. https://doi.org/10.25165/j.ijabe.20181104.4278

Shockley, J. M., Dillon, C. R., & Shearer, S. A. (2019). An economic feasibility assessment of autonomous field machinery in grain crop production. *Precision Agriculture*, *20*(5), 1068–1085. https://doi.org/10.1007/s11119-019-09638-w

Software Advice. (2021). *Timelines*. Retrieved July 07, 2021, from https://www.softwareadvice.com/scm/logistics-comparison/

Sørensen, C. G. (2003). A Model of Field Machinery Capability and Logistics: the case of Manure Application. *Agricultural Engineering International: The CIGR Journal of Scientific Research and Development*, *V*(October), Manuscript PM 03 004, pages 20. http://www.scopus.com/inward/record.url?eid=2-s2.0-84905920777&partnerID=tZOtx3y1

Sørensen, C. G., Madsen, N. A., & Jacobsen, B. H. (2005). Organic farming scenarios: Operational analysis and costs of implementing innovative technologies. *Biosystems Engineering*, *91*(2), 127–137. https://doi.org/10.1016/j.biosystemseng.2005.03.006

Sørensen, C. G., & Nielsen, V. (2005). Operational analyses and model comparison of machinery systems for reduced tillage. *Biosystems Engineering*, *92*(2), 143–155. https://doi.org/10.1016/j.biosystemseng.2005.06.014

Spekken, M., & de Bruin, S. (2013). Optimized routing on agricultural fields by minimizing maneuvering and servicing time. *Precision Agriculture*, *14*(2), 224–244. https://doi.org/10.1007/s11119-012-9290-5

Taylor, R. K., Schrock, M. D., & Staggenborg, S.A. (2001). Using GPS Technology to Assist Machinery Management Decisions. *ASAE Meeting Paper No. MC01-204. ASAE, 2950 Niles Road, St. Joseph, MI 49085-9659 USA.*, (January 2001).

Taylor R K., Schrock M D., & Staggenborg S A. (2002). Extracting Machinery Management Information from GPS Data. *Paper No. 02-10008. St. Joseph, MI: ASAE, 2002.*, (May). https://doi.org/10.13031/2013.13933

Witney, B. (1988). *Choosing and Using Farm Machines*. Longman Scientific & Technical, Edinburgh, Scotland, UK.

**SUPPLEMENTARY TABLES: TABLE S1 TO TABLE S3**

**Table S1** Assumptions of parameters used in the field size algorithms

| Operations | Equipment* | Equipment specifications** | | | | | | | | | | | Field specifications*** | | | |
| --- | --- | --- | --- | --- | --- | --- | --- | --- | --- | --- | --- | --- | --- | --- | --- | --- |
|  |  | Width of the implement | Interior  field speed | Turning speed | Turning radius | Overlap percentage | Input application rate | Bin capacity | Fill time | Fuel consumption rate | Tank capacity | Fill time | Effective swath width | Swath skipped | Width of the headland | Area |
|  | Unit | m | km/h | km/h | m | % | kg/ha | kg | min. | L/ha | L | min. | m | No. | m | m^2^ |
| Drilling | HFH | 1.5 | 3.25 | 1.0833 | 3.7 | 10 | 140 | 180 | 10 | 4 | 35 | 10 | 1.35 | 2 | 6.3 | *Rectangular Fields*: 10000; 100000; 200000; 500000; 750000; 1000000  *Right-angled Triangular Fields*:  10000; 100000; 200000; 250000 |
|  | Large Conventional | 6 | 5 | 1.6667 | 7 | 10 | 140 | 3000 | 20 | 8.82 | 463 | 20 | 5.4 | 0 | 32.4 |  |
|  | Small Conventional | 3 | 5 | 1.6667 | 4.35 | 10 | 140 | 2000 | 15 | 8.82 | 175 | 15 | 2.7 | 0 | 21.6 |  |
|  | Unit | m | km/h | km/h | m | % | L/ha | L | min. | L/ha | L | min. | m | No. | m |  |
| Spraying | HFH | 7 | 5 | 1.6667 | 3.7 | 10 | 200 | 1000 | 10 | 4 | 35 | 10 | 6.3 | 0 | 6.3 |  |
|  | Large Conventional | 36 | 10 | 3.3333 | 7 | 10 | 200 | 5000 | 20 | 8.82 | 463 | 20 | 32.4 | 0 | 32.4 |  |
|  | Small Conventional | 24 | 10 | 3.3333 | 4.35 | 10 | 200 | 2000 | 15 | 8.82 | 175 | 15 | 21.6 | 0 | 21.6 |  |
| Harvesting | HFH | 2 | 3.25 | 1.0833 | 3.7 | 10 | - | - | - | 4 | 35 | 10 | 1.8 | 2 | 6.3 |  |
|  | Large Conventional | 7.5 | 3 | 1 | 7 | 10 | - | - | - | 8.82 | 300 | 20 | 6.75 | 0 | 32.4 |  |
|  | Small Conventional | 4.5 | 3 | 1 | 7 | 10 | - | - | - | 8.82 | 300 | 20 | 4.05 | 0 | 21.6 |  |

**Note:** *HFH equipment refers identical 28 kW conventional machine with human operator and autonomous machine, large conventional machine refers 221 kW equipment with human operator, and small conventional machine indicates 112 kW machine with human operator. **HFH equipment characteristics were collected from the HFH demonstration project experience at Harper Adams University, Newport, Shropshire, United Kingdom. Large and small equipment (with human operator) specifications were collected from John Deere 6120M; John Deere 7R 270; John Deere T550; Arslan et al. (2014); and Lowenberg-DeBoer *et al.* (2021). *** The study assumed that length of the rectangular field is ten times the width of the field as length 316 m and 1000 m and width were 32, 100, 200, 500, 750, 1000 m for calibrating 1ha, 10 ha, 20 ha, 50 ha, 75ha, and 100 ha fields respectively. In addition, for different sized right-angled triangular fields (i.e., 1ha, 10ha, 20ha, and 25ha), where the height equalling twice the base. The 10 ha field was selected for the larger fields and 1 ha field is considered because relatively few fields in the United Kingdom are smaller than 1 ha. In addition, 1 ha fields also represents the smallholders’ in developing countries such as Asia and Africa, where the average field size is less than 2 hectares (High Level Panel of Experts (HLPE), 2013; Lowder et al., 2016).

**Table S2** Parameter definitions

| Parameters | Description | Unit |
| --- | --- | --- |
| A | area of the field | Square meter (m^2^) |
| $\boldsymbol{A}_{\boldsymbol{if}}$ | area of the interior field | m^2^ |
| $\boldsymbol{A}_{\boldsymbol{hf}}$ | area of the headland | m^2^ |
| $\boldsymbol{A}_{\boldsymbol{bf}}$ | adjacent base of the field | Meter (m) |
| $\boldsymbol{A}_{\boldsymbol{bif}}$ | adjacent base of the interior field | m |
| $\boldsymbol{C}_{\boldsymbol{b}}$ | capacity of the bin | Kilogram (kg) |
| $\boldsymbol{C}_{\boldsymbol{t}}$ | capacity of the fuel tank | Litre (L) |
| $\boldsymbol{E}_{\boldsymbol{f}}$ | field efficiency | Percentage (%) |
| $\boldsymbol{H}_{\boldsymbol{ypf}}$ | hypotenuse of the field | m |
| $\boldsymbol{IR}_{\boldsymbol{if}}$ | input required for the interior field | kg |
| $\boldsymbol{IR}_{\boldsymbol{h}}$ | input required for the headland | kg |
| $\boldsymbol{IR}_{\boldsymbol{hif}}$ | input required for the headland and interior field | kg |
| $\boldsymbol{L}_{\boldsymbol{f}}$ | length of the field | m |
| $\boldsymbol{L}_{\boldsymbol{if}}$ | length of the interior field | m |
| $\boldsymbol{l}_{\boldsymbol{pi}}$ | length of the interior field passes | m |
| $\boldsymbol{L}_{\boldsymbol{P}}$ | total length of the interior passes | m |
| n | number of swaths skipped during turning | Number (No.) |
| $\boldsymbol{n}_{\boldsymbol{h}}$ | number of rounds required for the headland operation | No. |
| N | total number of interior headlands turn | No. |
| $\boldsymbol{n}_{\boldsymbol{P}}$ | number of passes in the interior field operation | No. |
| $\boldsymbol{N}_{\boldsymbol{P}}$ | total number of interior passes | No. |
| $\boldsymbol{N}_{\boldsymbol{hp}}$ | number of passes around the headland | No. |
| $\boldsymbol{NRN}_{\boldsymbol{f}}$ | number of refills needed “within” the field | No. |
| $\boldsymbol{O}_{\boldsymbol{hf}}$ | opposite height of the field | m |
| $\boldsymbol{O}_{\boldsymbol{hif}}$ | opposite height of the interior field | m |
| $\boldsymbol{P}_{\boldsymbol{whhyp}}$ | projection of the width of the headland on the hypotenuse | m |
| $\boldsymbol{Q}_{\boldsymbol{s}}$ | seeding rate per ha | kg/ha |
| $\boldsymbol{Q}_{\boldsymbol{f}}$ | fuel consumption rate per ha | L/ha |
| r | turning radius of the implement | m |
| $\boldsymbol{T}_{\boldsymbol{hi}}$ | time required for the headland round | Hour (h) |
| $\boldsymbol{T}_{\boldsymbol{h}}$ | total time in the headland round | h |
| $\boldsymbol{T}_{\boldsymbol{P}}$ | total time in the interior field passes | h |
| $\boldsymbol{T}_{\boldsymbol{hturn}}$ | turning time from headland to first interior pass | h |
| $\boldsymbol{T}_{\boldsymbol{turn}}$ | interior headland turning time | h |
| $\boldsymbol{T}_{\boldsymbol{fe}}$ | total time for field entry and exit passes | h |
| $\boldsymbol{T}_{\boldsymbol{obs}}$ | total observed time in the interior field and passes | h |
| $\boldsymbol{T}_{\boldsymbol{r}}$ | total interior headlands turning time | h |
| $\boldsymbol{T}_{\boldsymbol{ssir}}$ | stoppage time for a single input refill | h |
| $\boldsymbol{T}_{\boldsymbol{sir}}$ | total stoppage time for input refill | h |
| $\boldsymbol{T}_{\boldsymbol{ssfr}}$ | stoppage time for single fuel refill | h |
| $\boldsymbol{T}_{\boldsymbol{sfr}}$ | total stoppages time for fuel refill | h |
| $\boldsymbol{T}_{\boldsymbol{sb}}$ | total stoppage time for blockage | h |
| $\boldsymbol{T}_{\boldsymbol{sf}}$ | total stoppages time “within” the field | h |
| $\boldsymbol{T}_{\boldsymbol{tfo}}$ | total time for field operation | h |
| $\boldsymbol{T}_{\boldsymbol{tfoha}}$ | total time for field operation per hectare | h |
| $\boldsymbol{T}_{\boldsymbol{T}}$ | theoretical field time | h |
| $\boldsymbol{T}_{\boldsymbol{Tha}}$ | theoretical field time per hectare | h |
| $\boldsymbol{v}_{\boldsymbol{p}}$ | implement running speed in the passes | Kilometre per hour (km/h) |
| $\boldsymbol{v}_{\boldsymbol{t}}$ | implement turning speed | km/h |
| $\boldsymbol{W}_{\boldsymbol{Ts}}$ | theoretical swath width | m |
| $\boldsymbol{W}_{\boldsymbol{f}}$ | width of the field | m |
| $\boldsymbol{W}_{\boldsymbol{h}}$ | Width of the headland | m |
| $\boldsymbol{W}_{\boldsymbol{if}}$ | width of the interior field | m |
| $\boldsymbol{W}_{\boldsymbol{m}}$ | width of the implement | m |
| $\boldsymbol{W}_{\boldsymbol{s}}$ | effective swath width | m |
| $\boldsymbol{W}_{\boldsymbol{h2ndlc}}$ | width of the headland on the second largest corner | m |
| $\boldsymbol{\delta}$ | swath direction | Radians (rad) |
| $\boldsymbol{\theta}$ | right angle of the right-angled triangular field | rad |
| $\boldsymbol{\varphi}$ | second largest angle | rad |
| $\boldsymbol{\mu}$ | smallest angle | rad |

**Table S3** Field efficiency comparisons subject to field shapes

| Field Operations | Equipment | Present Study (2021) * | | Hunt (2001) ** | Witney (1988) *** |
| --- | --- | --- | --- | --- | --- |
|  |  | Rectangular Field | Triangular Field |  |  |
| Drilling | HFH equipment | 89% | 70% | 77-90% | 75-85% |
|  | Small conventional equipment with human driver | 94% | 54% |  |  |
|  | Large conventional equipment with human driver | 87% | 43% |  |  |
| Spraying | HFH equipment | 69% | 66% | 55-80% | 55-65% |
|  | Small conventional equipment with human driver | 70% | 46% |  |  |
|  | Large conventional equipment with human driver | 65% | 36% |  |  |
| Harvesting | HFH equipment | 91% | 71% | 63-90% | 65-75% |
|  | Small conventional equipment with human driver | 83% | 48% |  |  |
|  | Large conventional equipment with human driver | 87% | 41% |  |  |

**Note:** *Authors estimation, assumed 10 ha rectangular and right-angled triangular field (See Supplementary Materials i.e., SM1 Rectangular Field Algorithms and SM2 Non-Rectangular Field Algorithms); ** See Table 1.1, p. 5, Hunt (2001); *** See Table 3.3, p. 103, Witney (1988).
